# All-in-one approaches for triple-negative breast cancer therapy: metal-phenolic nanoplatform for MR imaging-guided combinational therapy

**DOI:** 10.1186/s12951-022-01416-7

**Published:** 2022-05-12

**Authors:** Qi Xie, Shichao Li, Xingxing Feng, Jingyu Shi, Yang Li, Guanjie Yuan, Conglian Yang, Yaqi Shen, Li Kong, Zhiping Zhang

**Affiliations:** 1 Tongji School of Pharmacy, Huazhong University of Science and Technology, Wuhan, 430030 China; 2 Department of Radiology, Tongji Hospital of Tongji Medical College of Huazhong University of Science and Technology, Wuhan, 430030 China; 3 Liyuan Hospital of Tongji Medical College of Huazhong University of Science and Technology, Wuhan, 430030 China; 4 National Engineering Research Center for Nanomedicine, Huazhong University of Science and Technology, Wuhan, 430030 China; 5 Hubei Engineering Research Center for Novel Drug Delivery System, Huazhong University of Science and Technology, Wuhan, 430030 China

**Keywords:** Self-assembly, MPNs, Bleomycin, Chemodynamic therapy, MRI

## Abstract

**Background:**

Conventional chemotherapy has poor efficacy in triple-negative breast cancer (TNBC) which is highly heterogeneous and aggressive. Imaging-guided therapy is usually combined with diverse treatment modalities, could realize the integration of diagnosis and treatments. Therefore, the primary challenge for combinational therapy is designing proper delivery systems to accomplish multiple synergistic effects.

**Results:**

Herein, a facile nanoplatform was manufactured to fulfill the all-in-one approaches for TNBC combinational therapy. Fe^3+^-based metal-phenolic networks (MPNs) with bovine serum albumin (BSA) modification served as drug delivery carriers to encapsulate bleomycin (BLM), forming BFE@BSA NPs. The self-assembly mechanism, pH-responsive drug release behavior, and other physicochemical properties of this system were characterized. The potential of BFE@BSA NPs as photothermal transduction agents and magnetic resonance imaging (MRI) contrast agents was explored. The synergistic anti-tumor effects consisting of BLM-induced chemotherapy, Fenton reactions-mediated chemodynamic therapy, and photothermal therapy-induced apoptosis were studied both in vitro and in vivo. Once internalized into tumor cells, released BLM could cause DNA damage, while Fenton reactions were initiated to produce highly toxic •OH. Upon laser irradiation, BFE@BSA NPs could convert light into heat to achieve synergistic effects. After intravenous administration, BFE@BSA NPs exhibited great therapeutic effects in 4T1 tumor xenograft model. Moreover, as T_1_-weighted MRI contrast agents, BFE@BSA NPs could provide diagnosis and treatment monitoring for individualized precise therapy.

**Conclusions:**

A nano-system that integrated imaging and combinational therapy (chemotherapy, chemodynamic therapy and photothermal therapy) were developed to kill the tumor and monitor therapeutic efficacy. This strategy provided an all-in-one theranostic nanoplatform for MRI-guided combinational therapy against TNBC.

**Graphical Abstract:**

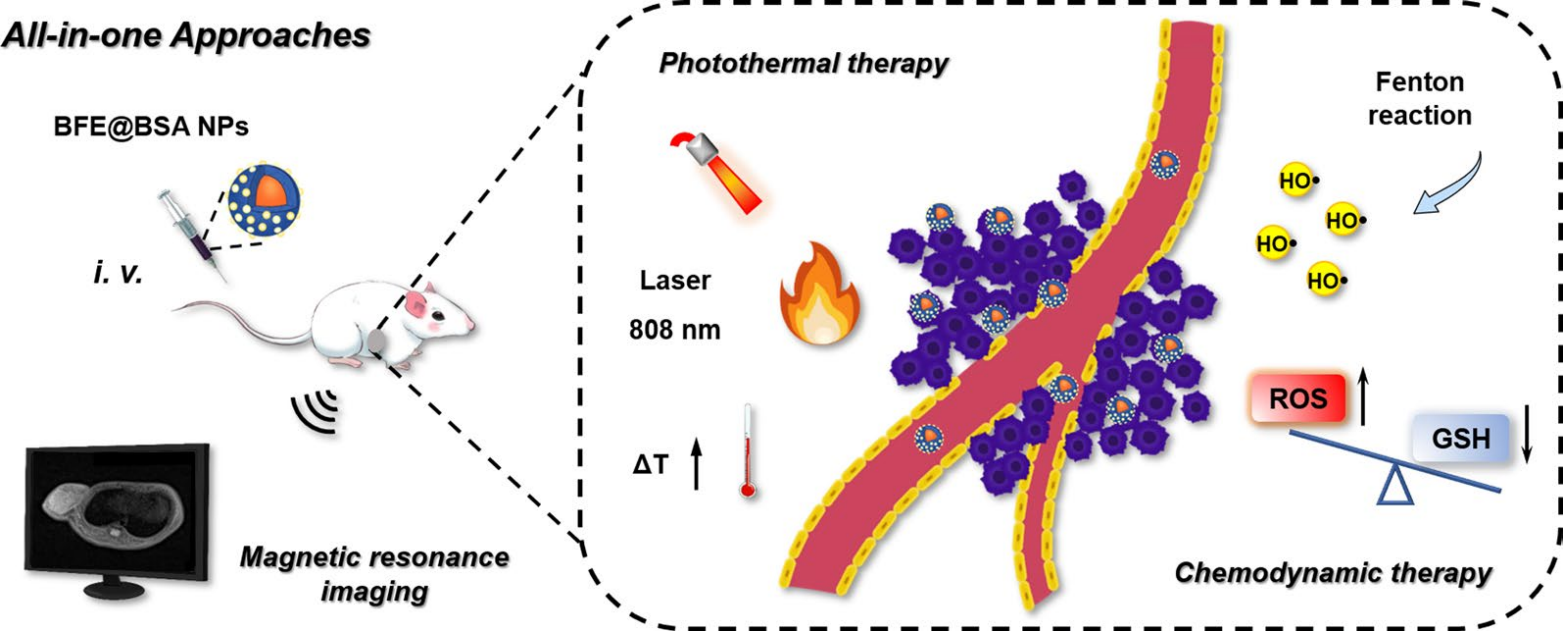

**Supplementary Information:**

The online version contains supplementary material available at 10.1186/s12951-022-01416-7.

## Introduction

The most common cancer among women is breast cancer, which is the second leading cause of cancer death in women after lung cancer [1, 2]. Triple-negative breast cancer (TNBC), which accounts for 15% of breast cancer, is highly heterogeneous and aggressive [3, 4]. TNBC is prone to recurrence and distant metastasis, resulting in an overall poor prognosis in patients [5]. Therefore, accurate tumor detection is essential to predict the patient’s response to treatment and guide treatment strategies, thereby improving the therapeutic effect of TNBC. For example, magnetic resonance imaging (MRI), as a diagnostic tool with noninvasiveness, high resolution and unlimited penetration depth [6], can provide real-time information on the pathological features and progression of tumors. Imaging-guided therapy has the potential to realize the integration of diagnosis and treatments. The current consensus among researchers is that monotherapy is far from adequate to achieve satisfactory therapeutic effects. Therefore, strategies of multiple combinational therapy assisted by imaging modalities have become the primary concerns.

Among the existing treatment strategies for TNBC, chemotherapy is still one of the main therapeutic options, although patients receiving chemotherapy tend to relapse more frequently and exhibit a worse prognosis [7–9]. As an alternative, dynamic therapy which could damage the cellular components by producing various reactive species such as reactive oxygen species (ROS), has become a promising cancer therapeutic strategy. Reactive species can not only arise from the stimulation of exogenous energy (e.g., light, ultrasound, and ionizing irradiation), but also from the activation of endogenous chemical energy sources such as hydrogen peroxide (H_2_O_2_) [10–13]. Chemodynamic therapy (CDT) is a specific cancer dynamic therapy that requires no external energy input. CDT initiates intracellular Fenton or Fenton-like reactions, converting H_2_O_2_ into strongly toxic hydroxyl radicals (•OH) under acidic tumor microenvironment (TME), thereby killing cancer cells. The overexpressed H_2_O_2_ in cancer cells serves as the reaction substrate, while transition metal elements such as iron and copper can be used as catalysts [14]. CDT is highly specific to the TME, and therefore exhibits better therapeutic effect and few side effects, compared with conventional chemotherapy [15, 16]. However, the mild acidity, low H_2_O_2_ content and overexpressed reducing substances such as glutathione (GSH) in TME sometimes limit the therapeutic efficacy of CDT [17].

It has been reported that the efficacy and sensitivity of cancer treatment of CDT would be significantly improved when combined with other therapies [18], such as increasing the temperature at tumor site by photothermal therapy (PTT) [19]. Elevated temperature could accelerate Fenton or Fenton-like reactions and promote the production of •OH. The rational combination of CDT and PTT can overcome the limitations and shortcomings of monotherapy to achieve synergistic therapeutic effects. Therefore, it is extremely important to design proper CDT reagents with photothermal transduction effects. Moreover, PTT is a powerful and non-invasive cancer treatment with high specificity and accurate spatio-temporal selectivity. Photothermal transduction agents (PTAs) are applied to capture and convert energy from light into heat for PTT, thereby raising the temperature of the local tissues to kill cancer cells [20, 21]. It causes less damage to surrounding healthy tissues and could eliminate a wide variety of tumors. In order to enhance therapeutic effect and reduce non-specific light damage to healthy tissues, PTAs are usually assisted by multiple imaging modalities to achieve precise treatment [22]. Reasonable design of therapeutic agents with imaging modalities could provide detailed information for tumor diagnosis and treatment monitoring, which offers new hope for effective TNBC treatment.

Metal ions are extensively applied in biomedical imaging. Iron is an essential trace metal element involved in many physiological processes of human body [23–25]. Recent studies have reported that iron ions and multidentate phenolic ligands (natural polyphenols) could form stable super-molecular network structures through coordination bonds, which are known as metal-phenolic networks (MPNs) [26–29]. Due to their intrinsic mechanical stability, thermal stability and pH responsiveness, MPNs have the potential to be excellent drug delivery vehicles with additional photothermal transduction ability and therapeutic functions [30–32]. For example, Fe^3+^ ions can serve as contrast agents for MRI on account of the significant T_1_ contrast enhancement effect. The magnetism of Fe^3+^ ions depends largely on their coordination state with the ligands [33–35]. MPNs possessed pH-responsive dissociation features, which enable MPNs to serve as T_1_-weighted contrast agents specific for weak acidic TME. Moreover, excessive iron can up-regulate intracellular ROS, inducing tumor cell apoptosis [23, 36]. Exogenous iron element may offer new approaches for effective tumor therapy. Thus Fe^3+^ ions based-MPNs are capable of serving as theranostic nanoplatforms which simultaneously integrates diagnostics and multiple therapies to realize personalized precise medicine [32, 37].

Bleomycin (BLM) is a class of glycopeptide antibiotics with potent antitumor activity. It is widely used in the clinical treatment of lymphomas, squamous cell carcinomas and germ cell tumors [38]. On account of the metal-binding domain, BLM could bind to transition metals (Fe^II^ or Cu^I^) and form BLM-metal complexes. In the presence of single-electron reductants and oxygen (O_2_), BLM-metal complexes were converted into activated forms, producing superoxide and hydroxyl radicals, that resulted in DNA cleavage and damage [39, 40]. However, BLM has severe dose-dependent pulmonary toxicity, which limits its clinical application. Pulmonary toxicity occurs in approximately 20% of patients treated with BLM, with pulmonary fibrosis being the most severe form [41]. Owing to its relatively large molecular weight (1.5 kDa) and hydrophilicity, BLM has very poor membrane permeability, resulting in low intracellular concentrations [42–45]. Hence, it is urgent to develop rational drug delivery systems for better therapeutic efficacy and lower side effects.

**Scheme 1:**
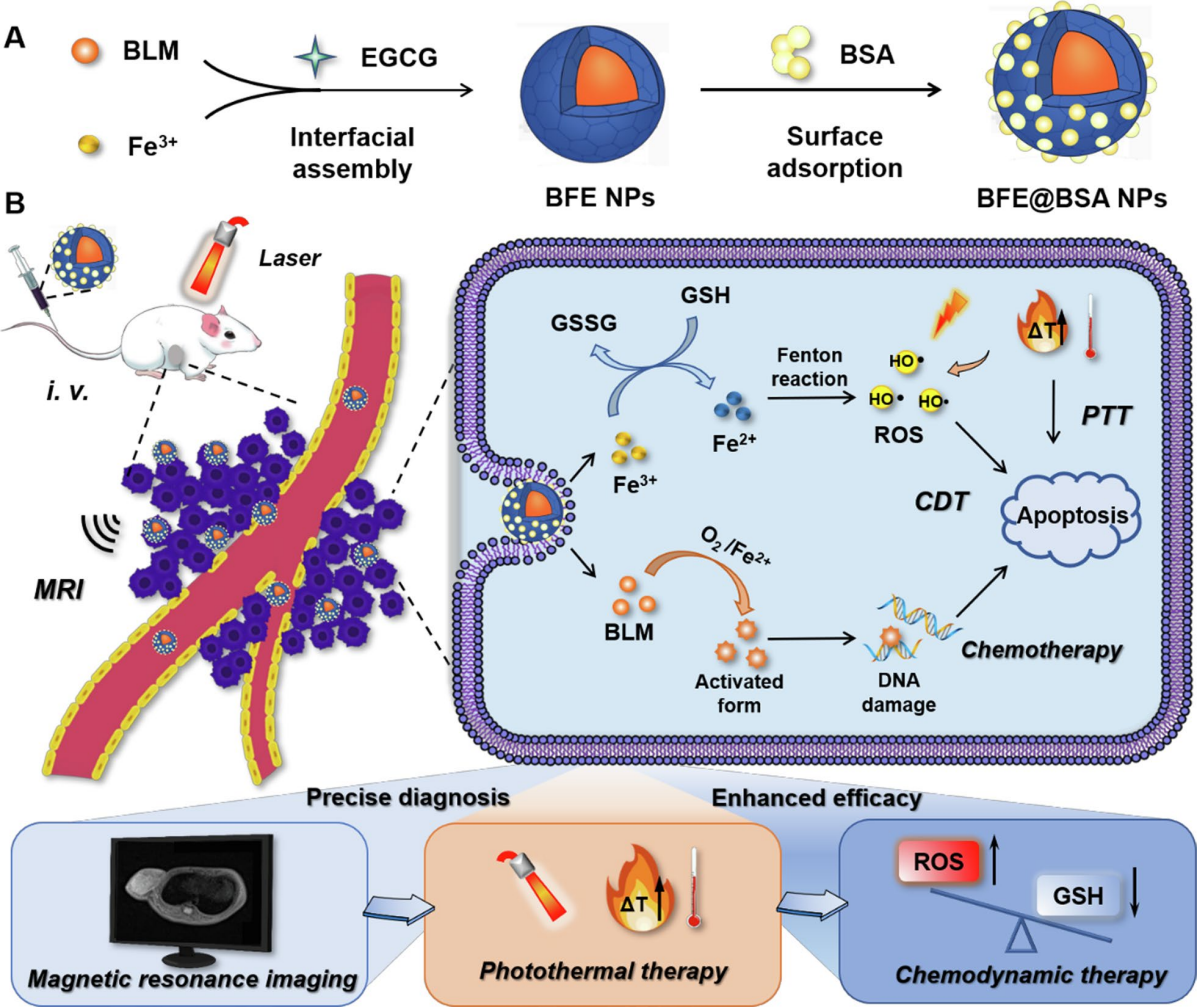
The assembly and combinational therapy mechanisms of BFE@BSA NPs. **A** The two-step assembly method to prepare BFE@BSA NPs. **B** The synergistic therapeutic effect of BFE@BSA NPs for TNBC

Herein, we proposed a MPNs-based nanoplatform to accomplish the all-in-one approach for TNBC combinational therapy (Scheme 1). On the whole, MPNs formed by epigallocatechin gallate (EGCG, tea polyphenol) and Fe^3+^ could effectively encapsulate BLM to prepare BFE NPs by self-assembly. In order to improve the stability of nanoparticles and mask the strong adhesion of polyphenols, which may lead to strong protein interactions in bloodstream [46, 47], bovine serum albumin (BSA) was coated on the surface of BFE NPs, forming BFE@BSA NPs. In our design, as T_1_-weighted MRI contrast agents, Fe^3+^ ions could provide diagnosis and treatment monitoring for individualized precise therapy. We further investigated both in vitro and in vivo therapeutic efficacy of BFE@BSA NPs on murine TNBC cell line (4T1) and tumor xenograft model. Once internalized into tumor cells, Fe^3+^ ions were reduced into Fe^2+^ ions by GSH. Then Fe^2+^ and BLM initiated Fenton reactions to produce highly toxic •OH, leading to GSH depletion and ROS amplification. As a chemotherapeutic agent, activated BLM could cause DNA damage. Upon laser irradiation, BFE@BSA NPs converted light into heat to ablate tumor. Furthermore, elevated local temperature contributed to accelerated Fenton reactions. This strategy provided an all-in-one theranostic nanoplatform for MRI-guided synergistic photothermal and chemodynamic therapy against TNBC.

## Experimental section

### Materials

BLM was purchased from Acmec biochemical (China). Bovine serum albumin (BSA) and EGCG were purchased from Macklin (China). FeCl_3_·6H_2_O was purchased from Aladdin (China). Phosphate buffer saline (PBS) was purchased from Servicebio (China). Penicillin–streptomycin solution (100  ×), fetal bovine serum (FBS), RPMI medium 1640, and trypsin were purchased from Gibco (USA). 4′,6-diamidino-2-phenylindole (DAPI), fluorescent dye 1,1′-dioctadecyl-3,3,3′-dio tetramethylindotricarbocyanine iodide (DiR) and ROS assay kit were obtained from Beyotime (China). Ethanol absolute, ammonia solution, ethylenediamine tetraacetic acid (EDTA), urea, glucose and all other chemical reagents were purchased from Sinopharm Chemical Reagent Co., Ltd (China).

### Cell culture and animal model

Murine TNBC cell line (4T1) was cultured with RPMI medium 1640 (containing 10% FBS, 100 IU/mL of penicillin and 100 μg/mL of streptomycin) in a humidified atmosphere incubator with 5% CO_2_ at 37 ℃. Female BALB/c mice (6 to 8 weeks) were purchased from the Laboratory Animal Resources of Huazhong University of Science and Technology (HUST) and raised under specific pathogen-free (SPF) conditions in the Animal Center of HUST. All animal experiments were reviewed and approved by the Experimental Animal Committee of HUST. 4T1 tumor xenograft model was established as follows: the suspension of 4T1 cells (2 × 10^5^ cells in 200 μL PBS) were inoculated under the flank skin of mice. The volume of tumors was calculated as V = L × W^2^/2 (L: length of tumor; W: width of tumor).

### Preparation and optimization of nanoparticles

#### Preparation of BFE NPs

Firstly, BFE NPs were prepared by BLM, FeCl_3_·6H_2_O and EGCG. BLM (0.3 mg) was dissolved in water (50 μL) and mixed with FeCl_3_·6H_2_O (0.3 mg) which was dissolved in water (15 μL) at room temperature. After stirring for 5 min, the mixture of BLM and FeCl_3_·6H_2_O was dispersed in ethanol absolute (1 mL) which contained 50 μL of EGCG solution (6 mg/mL). After stirring for 5 min, NH_3_·H_2_O (30 μL) was added to the mixture solution. Subsequently, the products were centrifuged (14,000 rpm) for 10 min at 4 ℃ to collect precipitation. The obtained precipitation (BFE NPs) was redispersed in 1 mL of water and sonicated for 1 min. To explore the optimal ratio of EGCG, the amount of BLM and Fe^3+^ was fixed, the preparation process was the same as the above method at different molar ratios (BLM:EGCG:Fe^3+^ = 1:X:6, X = 0, 1, 2, 3, and 4). Similarly, the amount of BLM and EGCG was fixed, the nanoparticles were prepared at different molar ratios (BLM:EGCG:Fe^3+^ = 1:3:Y, Y = 0, 2, 4, 6, and 8) to explore the optimal ratio of Fe^3+^.

#### In vitro stability study

In order to test the stability of nanoparticles in different media, BFE NPs were dispersed in ultrapure water (H_2_O), 5% glucose solution (Glu), normal saline (NS), phosphate buffer (PB), or PBS, respectively. After 24 h, the suspensions were photographed and measured. Furthermore, BFE NPs were dispersed in 100% FBS, DMEM (cell culture medium) or DMEM containing 10 or 50% FBS (denoted as 10 or 50% FBS-M), respectively. The long-term stability of nanoparticles was performed by storing the nanoparticles in ultrapure water, PBS and complete medium (RPMI medium 1640 containing 10% FBS, penicillin and streptomycin, denoted as 10% FBS-M) at 37 ℃ for 7 days. The particle size of nanoparticles was recorded at the corresponding time points.

#### Preparation of BFE@BSA NPs

In the following step, BFE NPs were further incubated in BSA solution (0, 1, 2, 5, 10 and 15 mg/mL) with gentle stirring for 1 h. The product was centrifuged at 14,000 rpm for 30 min (4 ℃) to remove free albumin and washed twice by repeated centrifugation. The precipitation was redispersed in 1 mL of water or PBS and sonicated for 1 min. The albumin-coated BFE NPs were denoted as BFE@BSA NPs.

### Characterization and spectra of nanoparticles

The hydrodynamic diameter, polydispersity index (PDI) and zeta potential of nanoparticles were measured by dynamic light scattering (DLS) by Zeta Potential Analyzer (Zeta PALS, Brookhaven Instruments, USA). Furthermore, the drug encapsulation efficiency (EE) of BLM and Fe^3+^ were detected by high performance liquid chromatography (HPLC) and atomic absorption spectrometry (AAS), respectively. Ultraviolet spectrophotometer (Lambda 365, PerkinElmer, USA), fluorescence spectroscopy analyzer (F-4600, Hitachi, Japan) and Fourier transform infrared spectroscopy (FT-IR) analyzer (AVATAR 360, Thermo, USA) were used for analyzing the properties and assembly mechanisms of BFE@BSA NPs. Transmission Electron Microscope (TEM, JEM-1230, Japan) was used to characterize the morphology of BFE NPs and BFE@BSA NPs.

### Self-assembly mechanisms study

BFE NPs and BFE@BSA NPs were dispersed in PBS, 0.9% NaCl solution, 5 M urea solution, 100 mM EDTA solution and 10% sodium dodecyl sulfate (SDS) solution, respectively. After 24 h, the suspensions were photographed and recorded. BFE@BSA NPs solution was treated with EDTA, and the fluorescence intensity of BFE@BSA NPs was recorded with excitation wavelength at 308 nm after different incubation.

### In vitro pH sensitive study

To further investigate the pH sensitivity of BFE@BSA NPs, 0.5 mL of BFE@BSA NPs was dispersed in 4 mL PBS with different pH values (7.4, 6.5 or 5.5). The fluorescence intensity of BFE@BSA NPs was recorded with excitation wavelength at 308 nm and the particle size of BFE@BSA NPs with different incubation time was also measured.

### In vitro drug release

To monitor the release of BLM from BFE@BSA NPs, the nanoparticles were suspended in 1 mL of PBS with pH values of 7.4, 6.5 and 5.5, respectively. All samples were further placed in a constant temperature vibration incubator at 37 ℃ (100 rpm). The supernatant was collected as the released medium at different time points by centrifugation at 10,000 rpm for 15 min. The BFE@BSA NPs precipitation was redispersed in equal volume fresh medium and kept at the same conditions. The content of BLM in release medium was determined by HPLC. The operation of experiment was repeated three times.

### In vitro photothermal evaluation

To assess the photothermal performance of BFE NPs and BFE@BSA NPs, the solution with different Fe^3+^ concentrations (0.2, 0.4 and 0.8 mM) was placed in a centrifuge tube (1.5 mL) to receive 808 nm laser irradiation with different power density of 2.0, 2.5, 3.0 W/cm^2^ for 10 min. The temperature variations were recorded using a digital infrared thermometer at designed time points.

### In vitro cytotoxicity assay

#### MTT assay

The cytotoxicity of BFE@BSA NPs was performed on 4T1 cell line. The cells were seeded in 96-well plates at a density of 1 × 10^4^ cells/well and cultured at 37 ℃ for 12 h. Free BLM and BFE@BSA NPs at different BLM concentrations (0, 1, 2, 4, and 8 μM) were added into cells and further incubated for another 6 h. Then, the cells were washed with PBS three times, the group of BFE@BSA NPs + L were irradiated under the 808 nm laser (2.5 W/cm^2^) for 2 min. After another 18 h, 10 μL of MTT (5 mg/mL) solution was added to each cell well and incubated for 4 h. Cell medium was removed before adding DMSO (150 μL) to dissolve the formazan crystals. To determine cell viability, absorbance at 490 nm was measured. The cell viability was calculated as follows:

$${\text{Cell}}\,{\text{viability}}\,\left( \% \right) = \left( {{\text{OD}}_{{{\text{sample}}}} - {\text{OD}}_{{{\text{blank}}}} /{\text{OD}}_{{{\text{control}}}} - {\text{OD}}_{{{\text{blank}}}} } \right) \times 100\%$$

#### Live/dead staining assay

The cells were seeded in 24-well plates at a density of 1 × 10^5^ cells/well and cultured at 37 ℃ for 12 h. Free BLM and BFE@BSA NPs at BLM concentration of 8 μM were added into cells and further incubated for another 6 h. Then, the cells were washed with PBS three times, the group of Laser and BFE@BSA NPs + L were irradiated under the 808 nm laser (2.5 W/cm^2^) for 2 min. After another 18 h, the live and dead cells were stained with calcein AM and PI according to the manufacturer’s instructions. The cells were then washed with cold PBS and observed by fluorescence microscope (CKX53, Olympus, Japan).

### In vitro cellular uptake study

Coumarin-6 loaded BFE NPs (BFE NPs_C6_) and BFE@BSA NPs (BFE@BSA NPs_C6_) were prepared for cellular uptake study, respectively. 4T1 cells were co-cultured with BFE NPs_C6_ or BFE@BSA NPs_C6_ (at coumarin-6 content of 0.5 μg/mL) for 2 h or 6 h. The nucleus was stained by DAPI after incubation. The cellular uptake images were acquired by fluorescence microscopy (CKX53, Olympus, Japan).

### In vitro ROS generation

4T1 cells were seeded in 6-well plates and cultured overnight. The medium was replaced with fresh medium containing free BLM (4 μM), BFE@BSA NPs (4 μM), or blank medium. After incubation for 8 h, the cells were washed with PBS three times. Then, the cells were irradiated under the 808 nm laser (2.5 W/cm^2^) for 15 s (this group was denoted as BFE@BSA NPs + L). The cells after different treatments were incubated with DCFH-DA at 37 ℃ for 30 min. Then, the cells were washed with PBS to remove free probe, following by observation via fluorescence microscopy and analysis via flow cytometry.

### In vitro MRI study of BFE@BSA NPs

The vitro MRI study of nanoparticles were carried out on a 3.0 T clinical MRI instrument (Skyra, Siemens Healthcare, Erlangen, Germany) with a Tx/Rx 15-channel knee coil.

#### In vitro relaxivity measurements

BFE@BSA NPs with different concentration gradients of Fe (0, 0.1, 0.2, 0.3, 0.4, and 0.5 mM) were prepared in water or agarose gel. The parameters for r_1_ measurements were set as follows: inversion time (TI) = 30, 60, 90, 120, 150, 250, 400, 600, 800, 1200, 1600, 2000, 2400, 2800, and 3200 ms; repetition time (TR) = 1500 ms + TI; echo time (TE) = 15 ms. The parameters for r_2_ measurements were set as follows: TR = 3000 ms; TE was between 20 and 600 ms with an interval of 20 ms. The following parameters were maintained for all measurements: slice thickness = 5 mm; field-of-view (FOV) = 120 × 120 mm. The relaxivity was calculated by the ImageJ and Sigma Plot software.

#### In vitro MRI performance

The series of BFE@BSA NPs solutions with different Fe concentrations were prepared and sealed in 2.0 mL Eppendorf tubes for further test. The experiments were carried out in both water and agarose gel. The parameters for T_1_‐weighted images (T_1_ WI) were set as follows: TR = 398 ms, TE = 14 ms, FOV = 120 × 120 mm^2^, matrix size = 256 × 256, slice thickness = 1 mm, and NEX = 8. The parameters for T_1_ mapping images (T_1_ Map) were set as follows: TR = 10.62 ms, TE = 3.7 ms, flip angle: 3–15° , number of signal averages = 4, FOV = 80 × 60 mm^2^, matrix size = 256 × 192, slice thickness = 1.5 mm and acquisition time = 1 min 30 s. T_1_ maps were automatically calculated pixel by pixel, the result displayed in multiple layers with visible color maps immediately after acquisition.

#### Cellular MR imaging

4T1 cells were seeded in 6-well plates at a density of 5 × 10^5^ cells/well and cultured overnight. Subsequently, the cells were co-incubated with or without BFE@BSA NPs at Fe^3+^ concentration of 1 mM. After incubation for 4 h, the cells were washed 3 times with PBS to eliminate the residual nanoparticles. Then, the cells were collected and resuspended in PBS (200 μL). Next, agarose gel (1%, 800 μL) was added to fix the cells. PBS was set as the blank control. The instrument and sequences were the same as the above method.

### In vivo MR imaging of mice

The in vivo MR images of tumors were acquired on a 3.0 T clinical MRI instrument (Skyra, Siemens Healthcare, Germany) with an 8-channel 5-cm Rx custom-design coil.

#### Experimental scheme

When the tumor volume grew to 300–500 mm^3^, female BALB/c mice bearing 4T1 tumors were anesthetized and then BFE@BSA NPs (1.1 mg Fe^3+^ per kilogram body weight) were injected through tail vein. T_1_‐weighted images and T_1_ mapping images were acquired at designed time points. The detailed parameters for in vivo T_1_ WI were set as follows: TR = 398 ms, TE = 17 ms, FOV = 40 × 40 mm^2^ (axial images) or 60 × 45 mm^2^ (coronal images), matrix size = 256 × 256, slice thickness = 1 mm, and NEX = 8. The parameters for T_1_ mapping images (T_1_ Map) were set as above. The mice were anesthetized with 1% isoflurane delivered via a nose cone during the imaging process.

#### Image analysis

The relative T_1_ signal and signal-to-noise ratio (SNR) were measured and calculated by two radiologists based on previous reports, using Equations (1) and (2), respectively:

1 $${\text{Relative}}\,{\text{T}}_{{1}} \,{\text{signal}}\,\left( \% \right) = {\text{S}}_{{{\text{Post}}}} /{\text{S}}_{{{\text{Pre}}}} \times 100\%$$

2 $${\text{SNR}} = {\text{S}}_{{{\text{Tumor}}}} /{\text{SD}}_{{{\text{Background}}}}$$

S_Pre_, S_Post_ and S_Tumor_ represented the signal intensity in the region-of-interest (ROI) placed on a homogeneously enhancing part of the tumor, SD_Background_ represented the standard deviation of the background noise [48].

### Biodistribution study of BFE@BSA NPs

DiR loaded BFE@BSA NPs (BFE@BSA NPs_DiR_) were prepared to evaluate biodistribution behavior. The 4T1 tumor-bearing mice were randomly divided into two groups (3 mice in each group). The mice were intravenously injected with 200 μL of free DiR or BFE@BSA NPs_DiR_ (0.5 mg DiR per kilogram body weight). After intravenous injection, the tumors and main organs were obtained and imaged (IVIS Lumina XR system, Trilogy, LI-COR) at designed time intervals.

### In vivo dose exploration of BLM

When the tumor volume grew to about 100 mm^3^, the 4T1 tumor-bearing mice were randomly divided into four groups (n = 5) and treated with BFE@BSA NPs at different BLM dosages (2.5, 5, and 10 mg per kilogram body weight). On the 5th, 8th and 11th day, mice were intravenously injected with 200 μL PBS or BFE@BSA NPs at different dosages. The body weight and tumor volume of each mouse were measured and recorded every other day. On the 21th day, all mice were sacrificed to harvest the tumors and major organs (heart, liver, spleen, kidneys, and lung), which were weighted and then preserved in 4% paraformaldehyde solution for histological analysis.

### In vivo anti-tumor combination therapy

The 4T1 tumor-bearing mice were randomly divided into four groups (n = 8) when the tumor volume grew to about 100 mm^3^. On the 6th, 9th and 12th day, mice were intravenously injected with 200 μL PBS or BLM, BFE@BSA NPs, BFE@BSA NPs + Laser (BFE@BSA NPs + L) at the same dosage (5 mg/kg). For the BFE@BSA NPs + L group, the tumor sites of mice were irradiated for 2 min at 12 h post-injection (808 nm laser at 2.5 W/cm^2^). The body weight and tumor volume of each mouse were measured and recorded every other day. On the 21st day, all mice were sacrificed to harvest the tumors and major organs (heart, liver, spleen, kidneys, and lung), which were weighted and then preserved in 4% paraformaldehyde solution for histological analysis.

### Statistical analysis

The data were presented in the form of mean ± standard deviation (SD). Statistical significance of experimental and control groups was analyzed by one-way ANOVA test at a significance level of p < 0.5 (*), p < 0.01 (**) and p < 0.001 (***).

## Results and discussions

### Preparation and optimization of nanoparticles

**Fig. 1:**
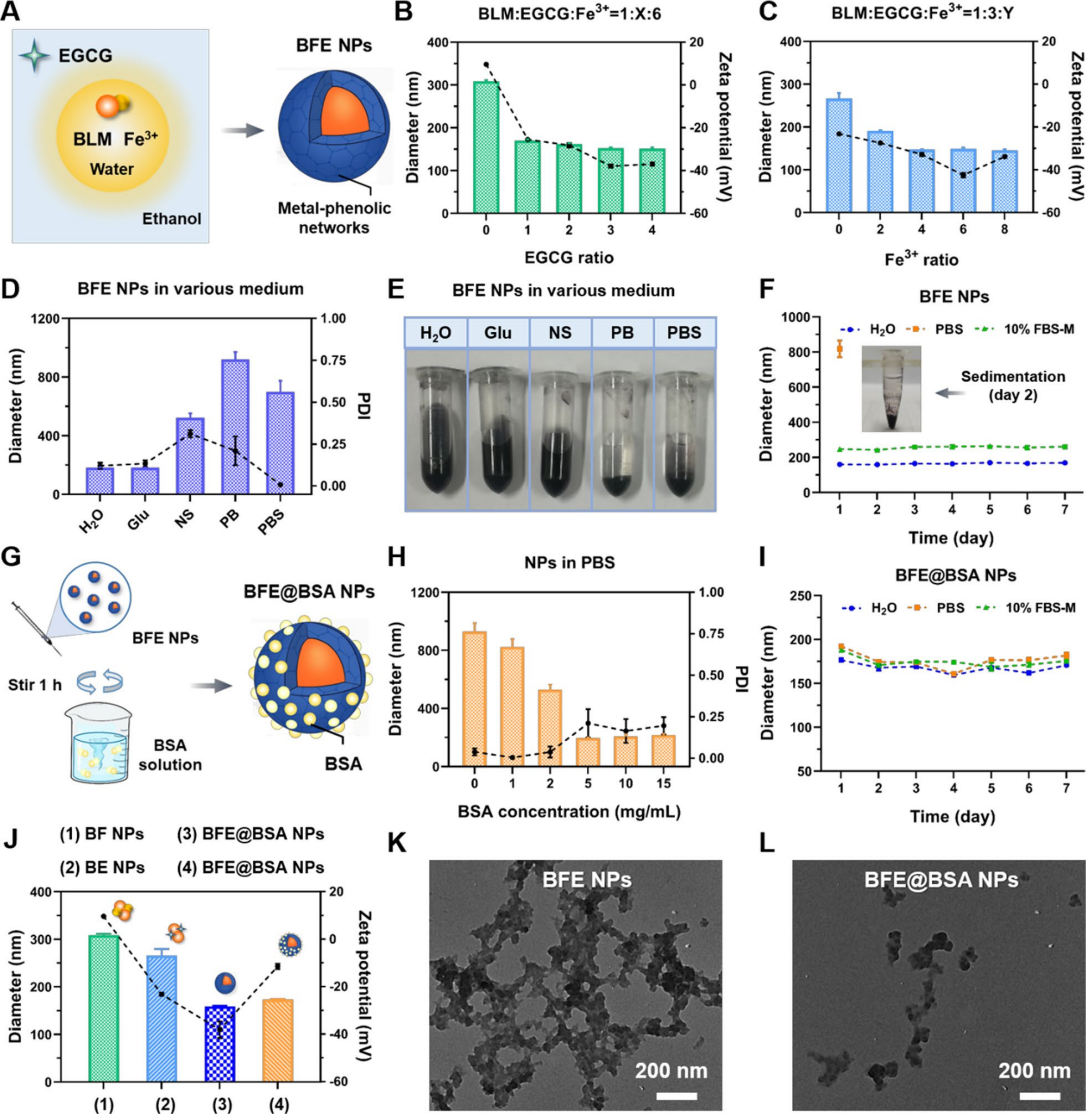
**A** Schematic description of BFE NPs preparation. The particle size and zeta potential of BFE NPs at different molar ratios of EGCG (**B**) and Fe^3+^ (**C**). The particle size, PDI (**D**) and photograph (**E**) of BFE NPs in various media. **F** The long-term stability of BFE NPs. **G** Schematic description of BFE@BSA NPs preparation. **H** The particle size and PDI of nanoparticles at different BSA concentrations. **I** The long-term stability of BFE@BSA NPs. **J** The summary of the four types of nanoparticles. The TEM images of BFE NPs (**K**) and BFE@BSA NPs (**L**). Scale bars were 200 nm

Even though EGCG and Fe^3+^ ions have been widely used to prepare MPNs, there were rare reports about using MPNs as carriers for delivery of BLM. Owing to the presence of metal-binding domain in BLM, Fe^3+^ ions could assemble with BLM in water, forming the core of nanoparticles [38, 39]. Then redundant Fe^3+^ ions further coordinated with EGCG at the interface of ethanol/water, composing MPNs (Fig. 1A and Additional file 1: Figure S1). We firstly investigated the proportion of three components required for the formation of BFE NPs. At beginning, the proportion of EGCG was explored by varying its input with fixed amount of BLM and Fe^3+^. As shown in Fig. 1B and Additional file 1: Figure S2A, when the molar ratio between BLM, EGCG and Fe^3+^ was set at 1:3:6, the corresponding nanoparticles exhibited the darkest color, whereas the particle size reached minimum and the zeta potential also decreased to the lowest. Similarly, the influence of the amount of Fe^3+^ on BFE NPs was examined. Using particle size and zeta potential as indicators, it has been further confirmed that the prepared nanoparticles were most suitable when the proportion of three components was 1:3:6 (Fig. 1C and Additional file 1: Figure S2B). Therefore, the molar ratio between BLM, EGCG and Fe^3+^ in the formation of BFE NPs was fixed at 1:3:6 in the following investigation.

The stability of nanoparticles is a prerequisite for subsequent application and storage. In order to simulate different biological conditions, the particle size changes of BFE NPs incubated in different media (H_2_O, Glu, NS, PB, and PBS) were monitored. After 24 h, BFE NPs were quite stable in both water and glucose solution, as the particle size of BFE NPs remained unchanged (Fig. 1D and E). But the particle size of BFE NPs increased exponentially in NS, PB and PBS, and sedimentation was even observed in PB and PBS. Furthermore, there was no precipitation of BFE NPs in 10 or 50% FBS-M or 100% FBS, and the particle size of nanoparticles slightly increased and then stayed steady (Additional file 1: Figure S3). Thus, we hypothesized that BFE NPs might adsorb proteins from the serum, which facilitated the stability of nanoparticles. In long-term stability study, BFE NPs were steady and remained the similar size for 7 days in the complete medium containing 10% FBS (Fig. 1F and Additional file 1: Figure S4A). In brief, BFE NPs aggregated in various salt solutions, but they exhibited colloidal stability in serum-containing solution, indicating that serum proteins might protect BFE NPs from aggregation.

Polyphenols are highly adherent and exhibit multiple interactions (e.g., hydrogen bonds, hydrophobic, and electrostatic interactions), which allow them to form robust conjugates with other substances. BSA has been widely used to modify nanoparticles to improve the stability. In order to improve the stability of BFE NPs, BFE NPs were further modified with BSA by incubation in BSA solution through surface adsorption (Fig. 1G). When the concentration of BSA reached 5 mg/mL (Fig. 1H), the nanoparticles could remain stable in PBS. Subsequently, with the concentration of BSA continually increased, the particle size of nanoparticles gradually increased. Therefore, the BSA concentration of 5 mg/mL was selected to prepare BFE@BSA NPs. As expected, the particle size of BFE@BSA NPs remained constant for 7 days in all media, including H_2_O, PBS and 10% FBS-M (Fig. 1I and Additional file 1: Figure S4B). The introduction of BSA endowed nanoparticles with great physiological stability and long-term stability, which laid a sufficient foundation for subsequent application.

To sum up, BLM + Fe^3+^ or BLM + EGCG could form nanoparticles (BF NPs or BE NPs). But only when three components were all present, nanoparticles with desired structure could be fabricated. As shown in Fig. 1J, BFE NPs which were prepared in optimal proportion displayed the particle size of 158.9 ± 1.8 nm and the zeta potential of − 38.1 ± 3.5 mV and presented a dark black appearance. The negative surface charge of BFE NPs indicated the structure of MPNs. After BSA coating on the surface of nanoparticles, BFE@BSA NPs remained the black appearance, while their particle size increased by ~ 15 nm (173.0 ± 1.4 nm) than BFE NPs and zeta potential was back to − 11.5 ± 1.1 mV. As a result of the masking of BSA, BFE@BSA NPs were less charged, which remarkably improved the stability of nanoparticles.

### Characterization and spectra of nanoparticles

**Fig. 2:**
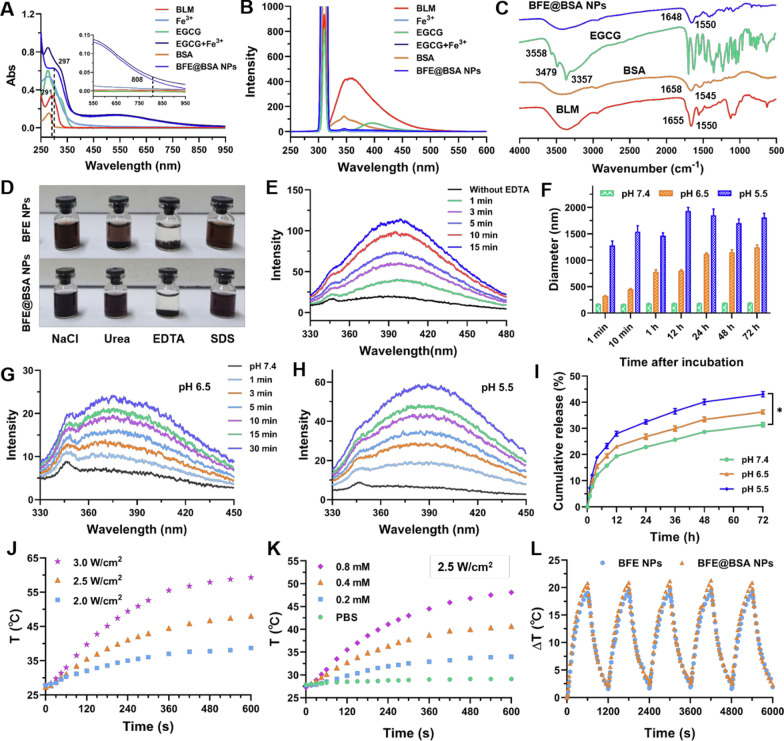
The UV–vis spectra (**A**), fluorescent spectra (**B**) and FT-IR spectra (**C**) analysis. **D** The photographs of BFE NPs and BFE@BSA NPs dissociation by NaCl, Urea, EDTA and SDS. **E** The fluorescence recovery analysis of BFE@BSA NPs after treatment with EDTA at different time points. **F** The changes of particle size of BFE@BSA NPs at different pH conditions over 72 h. The fluorescence recovery analysis of BFE@BSA NPs in PBS at pH 6.5 (**G**) and pH 5.5 (**H**). **I** The drug release behavior of BLM in PBS at different pH value. **J** The temperature elevation of BFE@BSA NPs under 808 nm laser irradiation with different power density for 10 min at Fe^3+^ concentration of 0.8 mM. **K** The temperature elevation of BFE@BSA NPs under laser irradiation (808 nm, 2.5 W/cm^2^) for 10 min at different Fe^3+^ concentrations. (Note: The trend of 0.8 mM is the same one of 2.5 W/cm^2^ in Fig 2J) **L** The temperature changes of BFE@BSA NPs within 5 laser-on and off cycles. Statistical p values: *p < 0.05

The morphology of nanoparticles was studied by TEM. As shown in Fig. 1K–L and Additional file 1: Figure S5, BFE NPs possessed network-like structures, which indicated the successful formation of MPNs. While the images of BFE@BSA NPs presented scattered particles because BSA facilitated the dispersion of nanoparticles. As shown in Additional file 1: Table S1, the EE of BLM and Fe^3+^ in BFE@BSA NPs was 65.0% and 77.2% (80.4% and 94.9% in BFE NPs), respectively, indicating this delivery system realized efficient drug packaging. In order to confirm the formation of nanoparticles, the UV–vis spectra, fluorescent spectra and FT-IR spectra were subsequently applied to analyze the formation mechanism. In Fig. 2A, the maximum absorption wavelength of BLM at 291 nm was red-shifted to 297 nm in the UV–vis spectrum of BFE@BSA NPs, which manifested the π-π interactions between BLM, EGCG and BSA. Furthermore, BFE@BSA NPs and direct mixture of EGCG + Fe^3+^ exhibited broad absorption in the NIR window, confirming that Fe^3+^-based MPNs had the ability to serve as photothermal reagents. In Fig. 2B, BLM exhibited a fluorescence emission from 330 to 480 nm with excitation wavelength at 308 nm in its fluorescent spectrum. However, the fluorescence of BLM was quenched in BFE@BSA NPs as a result of the formation of coordination bonds between EGCG and Fe^3+^. In Fig. 2C, the -OH stretching bands (3556, 3479 and 3357 cm^−1^) of pure EGCG were not observed in the FT-IR spectrum of BFE@BSA NPs, demonstrating the formation of coordination bonds. BFE@BSA NPs exhibited the characteristic amide I and amide II band vibrations (1648 and 1550 cm^−1^) of BLM and BSA, indicating the existence of BLM and BSA in nanoparticles. All of the above results proved the successful preparation of BFE@BSA NPs.

### Self-assembly mechanism study of BFE NPs and BFE@BSA NPs

In order to further confirm the self-assembly mechanism of BFE NPs and BFE@BSA NPs, the prepared nanoparticles were dispersed into different solutions (NaCl, urea, EDTA and SDS) to determine the role of electrostatic force, hydrogen bonds, coordination bonds and hydrophobic force, respectively. In Fig. 2D, when BFE NPs were dispersed into 0.9% NaCl solution (electrostatic force-eliminating agents), a small amount of precipitation was observed, indicating there were certain electrostatic interactions within nanomedicines. Changes and precipitation were more obvious when BFE was mixed with urea solution which could deconstruct hydrogen bonds, suggesting the hydrogen bonds were involved in the formation of BFE NPs. However, it was found that SDS did not affect nanoparticles, suggesting hydrophobic force barely existed during the assembly process of BFE NPs. The most obvious change of BFE NPs was observed in appearance, where precipitate formed immediately, upon mixing with EDTA solution (coordination bonds-eliminating agents). These results implied that EDTA was the most effective treatment to disintegrate the BFE NPs and coordination force played the main force in the formation of BFE.

To sum up, we could speculate different forces that participated in the formation of nanoparticles as follow: coordination bonds > hydrogen bonds > electrostatic force ≫ hydrophobic force. Furthermore, when the BFE@BSA NPs were placed in the above solution, the changes were smaller than that of BFE NPs in appearance. This indicated that the presence of BSA could improve the stability of nanoparticles to a certain degree. It has been proved that the fluorescence of BLM in BFE@BSA NPs was quenched, owing to coordination between BLM and Fe^3+^ (Fig. 2B). Therefore, we further investigated the fluorescence recovery ability of BLM from BFE@BSA NPs by adding EDTA solution. The degradation of BFE@BSA NPs was supposed to induce the release of BLM, leading to fluorescence recovery. As shown in Fig. 2E, upon adding EDTA, the fluorescence of BLM gradually recovered and fluorescence intensity gradually increased with incubation time. It indicated that EDTA could destroy the structures of BFE@BSA NPs and lead to the release of BLM. It further confirmed that coordination force played a vital role in the self-assembly process of nanoparticles.

### In vitro pH sensitive study

The BFE@BSA NPs was designed with the characteristics of pH-responsive degradation in an acidic environment, owing to the pH responsiveness of coordination force and hydrogen bonding. In acidic conditions, the disassemble of BFE@BSA NPs was accompanied by BLM release in the acidic TME. To evaluate the pH-responsive capability of BFE@BSA NPs, the size distribution of nanomedicines was measured firstly through dispersing them into buffer solution with pH at 6.5 and 5.5. From the results in Fig. 2F, with the extension of incubation time, the particle size of nanoparticles increased gradually in an acidic environment. The particle size of BFE@BSA NPs remained steady with negligible changes at pH 7.4. But at pH 6.5 and 5.5, BFE@BSA NPs became larger than before, indicating that the nanoparticles were pH-responsive.

Furthermore, the fluorescence recovery ability of BLM from BFE@BSA NPs under acidic conditions (pH 6.5 and 5.5) was also investigated. As expected, the fluorescence intensity of BLM increased gradually with incubation time at both pH values in Fig. 2G and H. Moreover, the fluorescence intensity of BLM recovered more quickly at pH 5.5 than that at pH 6.5. These results implied that BFE@BSA NPs could gradually dissociate and release the BLM in an acidic environment.

### In vitro drug release

To accurately access the BLM release behavior from BFE@BSA NPs in the physiological condition, tumor sites and intracellular acidic environment, in vitro drug release tests were performed in PBS solutions with pH 7.4, pH 6.5 and pH 5.5, to simulate certain conditions. As shown in Fig. 2I, the amount of BLM released from BFE@BSA NPs was gradually increased as the pH of releasing medium decreased from 7.4 to 5.5. At pH 7.4, the amount of BLM released within the entire experimental period (72 h) was around 30%, while BLM released was up to 43% in PBS solution at pH 5.5. The pH sensitive release profile was mainly ascribed to the dissociation of the coordination bands in BFE@BSA NPs in acidic environment.

### In vitro photothermal evaluation

In our previous study, it was found that Fe^3+^ ions-based MPNs exhibited a broad absorption in the NIR region, which indicated that the designed reagents might have a photothermal transduction effect. To test the photothermal conversion efficiency of BFE@BSA NPs, the temperature differences (ΔT) of BFE@BSA NPs solution was checked under different Fe^3+^ concentrations or power densities. In Fig. 2J, K and Additional file 1: Figure S6A, B, with the increase of laser intensity (from 2.0 to 3.0 W/cm^2^) or iron ions concentration (from 0.2 to 0.8 mM), the temperature of BFE@BSA NPs solution increased significantly. The temperature of BFE@BSA NPs with Fe^3+^ concentration at 0.8 mM could increase up to 48.1 ℃ with temperature change about 21 ℃ after irradiation for 10 min (808 nm laser at 2.5 W/cm^2^). These results suggested that BFE@BSA NPs could serve as effective PTAs.

To further investigate the photothermal stability of nanomedicines, BFE NPs and BFE@BSA NPs (0.8 mM) were irradiated for 5 cycles of laser on (808 nm laser at 2.5 W/cm^2^, 10 min) and laser off (10 min), respectively. After 5 cycles, the ΔT between adjacent peaks was within 0.5 ℃ according to Fig. 2L. It indicated the excellent photothermal stability of both BFE NPs and BFE@BSA NPs under repeated NIR laser irradiation, while coating with BSA did not affect the photothermal conversion efficiency of nanoparticles.

### In vitro cytotoxicity assay

**Fig. 3:**
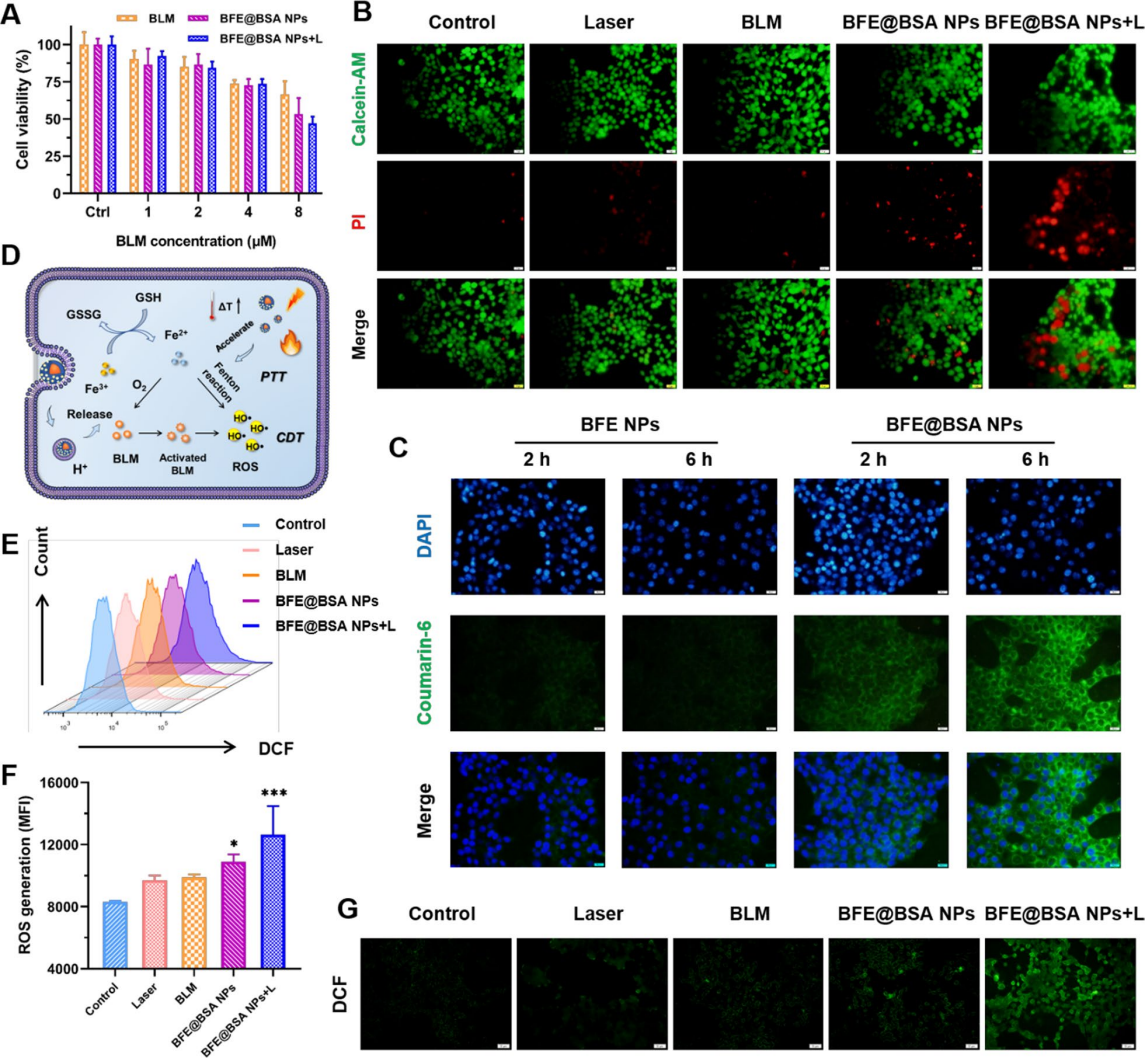
**A** The viability of 4T1 cells after different treatments (n = 6). **B** 4T1 cells stained by Calcein-AM/PI kit after different treatments. Scale bars were 20 μm. **C** The cellular uptake of coumarin-6 labeled BFE NPs and BFE@BSA NPs. Scale bars were 20 μm. **D** Schematic illustration of intracellular mechanisms. **E** The flow cytometry analysis of ROS generation after different treatments and (**F**) corresponding MFI values (n = 3). **G** The fluorescence images of ROS probes after different treatments. Scale bars were 50 μm. Statistical p values verse the control group: *p < 0.05, **p < 0.01, ***p < 0.001

The cell viability was firstly determined by MTT assay to investigate the in vitro anti-tumor ability of BFE@BSA NPs. 4T1 cells were incubated with free BLM or BFE@BSA NPs at different BLM concentrations. As shown in Fig. 3A, both free BLM and BFE@BSA NPs showed weak cytotoxicity at low concentrations. The cell viability of BFE@BSA NPs at BLM concentration of 8 μM was 53.2%, which was lower than that of free BLM (66.5%). And the cell viability of BFE@BSA NPs + L (BLM: 8 μM) was 47.1%, indicating that BFE@BSA NPs could efficiently kill tumor cells with laser irradiation. Then, live/dead staining assay was conducted by staining cells with Calcein-AM and PI after respective treatments. Calcein-AM (green) could easily penetrate live cell membrane to mark live cells, while PI (red) could only reach the cell nucleus through the disordered membrane of dead cell. As shown in Fig. 3B, the cells which were treated with laser alone showed almost no apparent red fluorescence. More red fluorescent signals were found in the groups treated with free BLM or BFE@BSA NPs (BLM: 8 μM), and cells treated with BFE@BSA NPs + L presented obvious red fluorescence. All the results illustrated the synergistic cytotoxicity of combination therapy.

### In vitro cellular uptake study

It was reported that the cellular uptake of nanomedicines was significantly influenced by their surface physicochemical characteristics. In order to understand whether the presence of BSA in our system would facilitate the cellular uptake, we evaluated the effect of BSA modification on the in vitro cellular uptake. Before that, green fluorescent dye, coumarin-6, was loaded into BFE NPs during the preparation for fluorescent observations. The internalization of BFE NPs and BFE@BSA NPs into 4T1 cells was observed by fluorescence microscopy. The blue area was the nuclei of 4T1 cells stained by DAPI and the green fluorescence resulted from coumarin-6 loaded nanoparticles. As shown in Fig. 3C, the amount of phagocytosed nanoparticles, no matter BFE NPs or BFE@BSA NPs, both increased with time, as stronger green fluorescence was observed at 6 h than that at 2 h, indicating the internalization of nanoparticles occurred in a time-dependent manner. What’s more, after incubation with BFE NPs or BFE@BSA NPs, the cytoplasm of 4T1 cells presented different intensities of coumarin-6 fluorescence. The fluorescence of cells treated with BFE@BSA NPs was much stronger than that in BFE NPs group, indicating that the surface modification of BSA could promote cellular uptake of nanoparticles.

### In vitro ROS generation

It is a truism that higher than the physiological level of ROS would trigger the damage of proteins, organelles and nucleic acids, thus leading to cell apoptosis. Excessive production of ROS within cancer cells, such as superoxide anion radical (O_2_^●−^) and hydroxyl radical (•OH), is often considered to be a significant condition for killing tumor cells. The designed system was supposed to produce excess ROS in multiple directions (Fig. 3D). In combination with Fe^II^, BLM was transformed into an activated form and could convert oxygen into hydrogen peroxide (H_2_O_2_). Highly toxic hydroxyl radicals were produced by the Fenton reactions under the high concentration of H_2_O_2_, leading to ROS amplification. Moreover, the transformation between Fe^3+^ ions and Fe^2+^ ions consumed glutathione heavily. This kind of GSH depletion could protect generated ROS from scavenging. Elevated temperature caused by PTT could further promote the production of ROS. Therefore, we evaluated the ROS generation ability of BFE@BSA NPs by incubation with 4T1 cancer cells. According to the fluorescence changes of a ROS-sensitive probe, 2′,7′-dichlorofluorescin diacetate (DCFH-DA), the amount of generated ROS could be confirmed. As shown in Fig. 3G, weak green fluorescence (DCF) was observed in control group or cells treated with laser, indicating that ROS produced by the metabolism of cells themselves was limited. The ROS level in 4T1 cancer cells treated with BLM alone increased slightly. It was reasonable since BLM could efficiently promote the formation of hydrogen peroxide only when catalyzed by adequate iron ions. Treating 4T1 cells with BFE@BSA NPs could facilitate the generation of ROS, as an increased fluorescence signal was observed in the cytoplasm. Upon laser irradiation, bright fluorescence was observed, indicating that the significantly enhanced ROS amplification effect was triggered by NIR. Furthermore, similar results were mirrored by flow cytometry analysis which were presented in Fig. 3E and F. These results implied that BFE@BSA NPs combined with PTT could synergistically induce ROS amplification.

### In vitro MRI study of BFE@BSA NPs

**Fig. 4:**
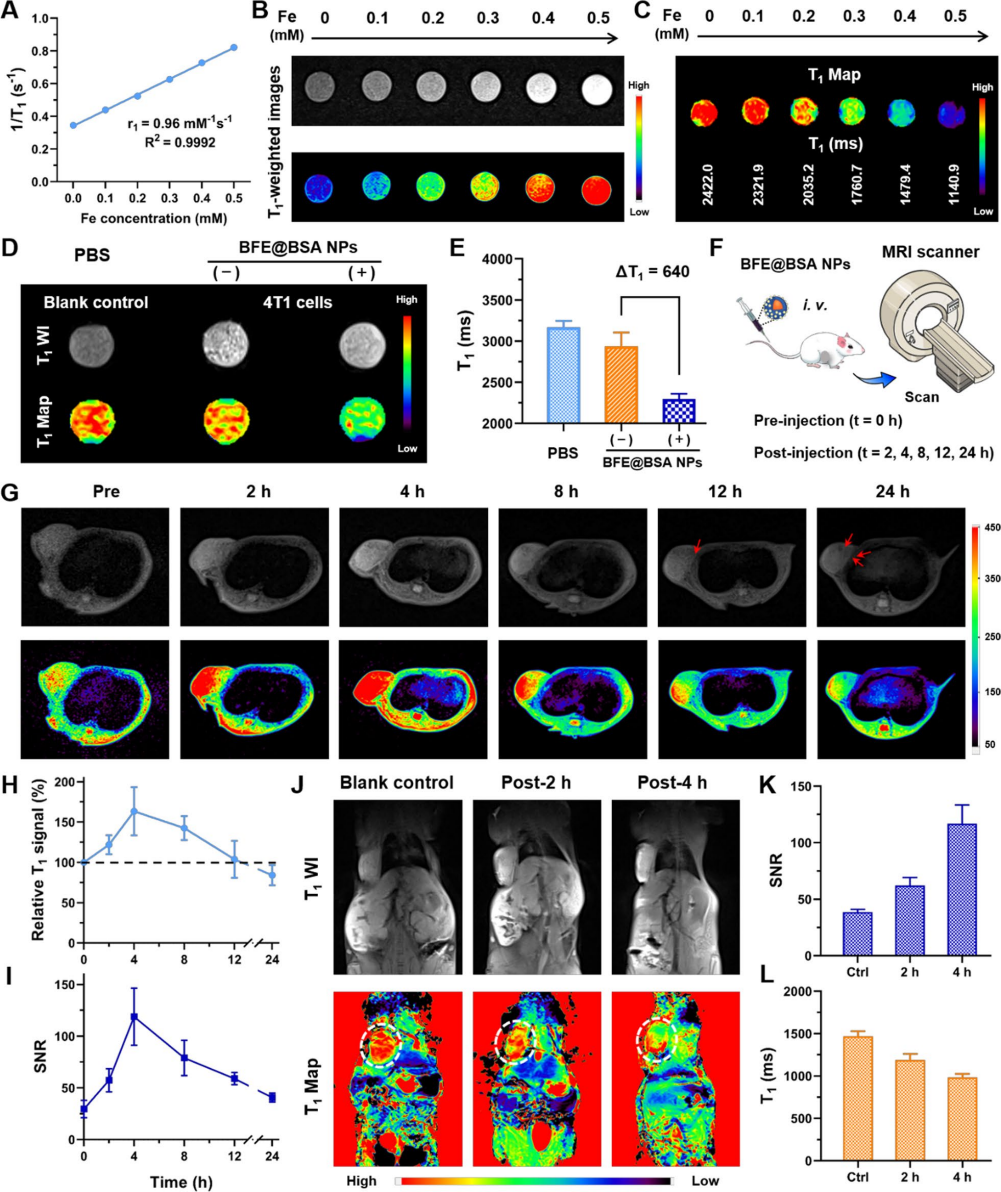
**A** The linear fitting of 1/T_1_ of BFE@BSA NPs at different Fe^3+^ concentrations. The T_1_-weighted MR images (**B**) and T_1_ mapping images (**C**) of BFE@BSA NPs nanoparticle in vitro. **D** The T_1_ WI and T_1_ Map of BFE@BSA NPs nanoparticle for cellular imaging. **E** The T_1_ value of each group (n = 3). **F** Schematic description of in vivo MR scanning plan. **G** The axial T_1_ WI of mice at different time points. The quantitative analysis of relative T_1_ signal (**H**) and SNR (**I**) in tumor region (n = 6, the same mouse for continuous monitoring). **J** The coronal T_1_ WI and T_1_ Map of mice at different time points. The quantitative analysis of SNR (**K**) and T_1_ value (**L**) in tumor region (n = 3, different mice for each time point). **F** The in vivo biodistribution of the DiR-loaded BFE@BSA NPs (n = 3). **G** The quantitative analysis of fluorescence intensity in tumor

The incorporation of Fe^3+^ ions enbaled BFE@BSA NPs to be potential MRI contrast agents. To further evaluate the contrast efficacy of BFE@BSA NPs, their longitudinal relaxivity (r_1_) and transverse relaxivity (r_2_) were tested firstly by scanning BFE@BSA NPs with different concentration gradients of Fe (0, 0.1, 0.2, 0.3, 0.4, and 0.5 mM). According to the graph data (Fig. 4A and Additional file 1: Figure S7), BFE@BSA NPs exhibited the r_1_ value of 0.96 mM^−1^ s^−1^ in water. The r_2_ value of BFE@BSA NPs was determined to be 2.41 mM^−1^ s^−1^, which was too low for BFE@BSA NPs to perform as T_2_-weighted contrast agents. But r_2_/r_1_ = 2.51 < 3, indicating BFE@BSA NPs were potential T_1_-weighted contrast agents for further application. [49] In Fig. 4B, it showed the T_1_-weighted images of BFE@BSA NPs at different Fe^3+^ concentrations, which presented positive bright contrast increasing. The MR signal was enhanced linearly with the increasing concentrations of Fe^3+^. BFE@BSA NPs dispersed in agarose gel also showed the obvious results (Additional file 1: Figure S8). The T_1_ value was measured by the T_1_ mapping images. As shown in Fig. C, BFE@BSA NPs could significantly shorten T_1_ relaxation time both in water and agarose gel, indicating they have good MR imaging ability in vitro. Then, BFE@BSA NPs were used for cellular MR imaging. [50] After incubation with nanoparticles for 4 h, the T_1_ value of 4T1 cells treated with BFE@BSA NPs was found to be significantly lower than that of cells without nanoparticles (Fig. 4D, E), indicating BFE@BSA NPs had the potential for further in vivo MR imaging.

### In vivo MR imaging of mice

It is extremely important to accurately monitor the size and location of tumor in the course of tumor therapy. Especially for photothermal or photodynamic therapy, accurate imaging of tumors can guide the course of treatment, monitor therapeutic efficacy, and reduce unexpected damage to normal tissues. MRI has become a diagnostic and research tool in treating various tumors because of its ability of accurately delineating the detailed images of the tumor tissue. As shown in Fig. 4F, 4T1 tumor-bearing BALB/c mice were i.v. injected with BFE@BSA NPs to assess in vivo MR imaging ability. In Fig. 4G, compared to pre-injection, the tumor region of mice was significantly brighter after injection of BFE@BSA NPs, owing to the effective accumulation of BFE@BSA NPs at the tumor site. The contrast between the tumor tissue and surrounding normal tissue was more pronounced, making tumor boundary clearer. In Fig. 4H, BFE@BSA NPs was continuously enriched at the tumor site and the T_1_ MR signal intensity gradually increased. At 4 h post-injection, the relative T_1_ signal of tumor site reached the maximum, which was about 1.63 times higher than that of pre-injection. After that, the MR signal intensity gradually decreased, or even fell below the value of pre-injection since 12 h post-injection. The quantitative analysis of SNR in tumor region presented the similar results (Fig.4I). It might be caused by the increased tumor necrotic areas (red arrow represented the necrosis of tumor tissue), which indicated the cytotoxic effect of BFE@BSA NPs upon arrival of the tumor region. [51] From all these results, it could reflect that effective accumulation of BFE@BSA NPs at the tumor sites could favor the precise MR imaging and cause necrosis of the tumor tissue, facilitating further therapeutic applications.

Based on the above axial images, coronal MR scanning was performed to study various organs of the mice. Furthermore, the coronal T_1_-weighted MR images and T_1_ mapping images of mice showed the similar trend. As shown in Fig. 4J, after intravenous injection of BFE@BSA NPs, the tumor became brighter over time compared to other organs, and the average SNR of tumor gradually increased (Fig. 4K). In T_1_ mapping images, BFE@BSA NPs visibly decreased the T_1_ value of tumor, the T_1_ relaxation time was gradually shortened (Fig. 4L). In Additional file 1: Figure S9, the tumor and other organs could be clearly distinguished (the organs were marked with different colored arrows), and the SNR of different organs increase over time. Although the SNR of different organs increased over time, the relative signal ratio of tumor to other organs also increased, indicating that BFE@BSA NPs exhibited good enhancement effects on the signal of tumor.

### Biodistribution study of BFE@BSA NPs

**Fig. 5:**
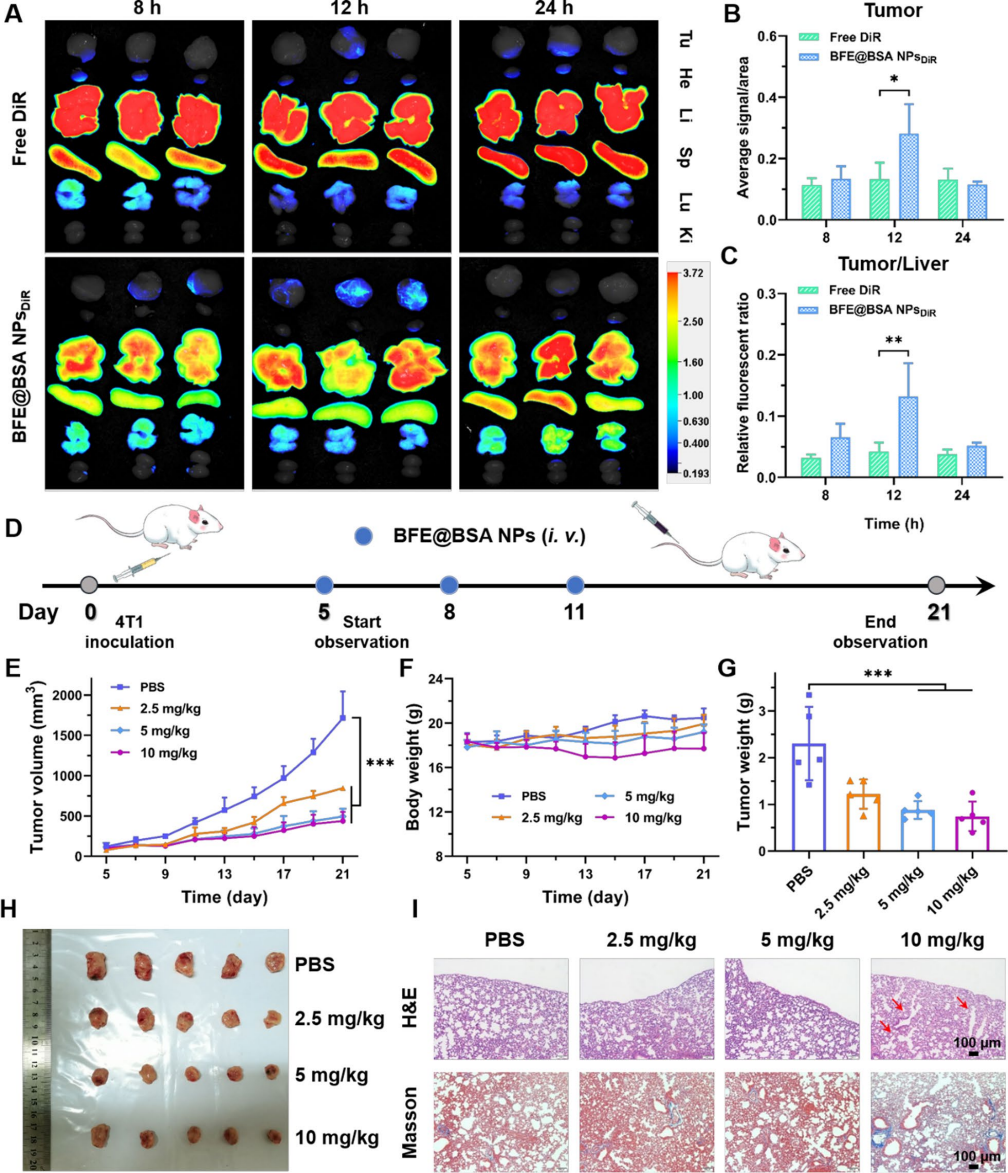
**A** The in vivo biodistribution of the DiR-loaded BFE@BSA NPs (n = 3, different mice for each time point). The quantitative analysis of fluorescence intensity of tumor (**B**) and relative fluorescent ratio of tumor/liver (**C**). **D** Schematic diagram of 4T1 tumor treatment. The 4T1 tumor growth curve (**E**), body weight of mice (**F**) and tumor weight (**G**) after different treatments (n = 5). **H** The photograph of tumors after different treatments. **I** H&E and masson’s trichrome staining of the lung sections from the mice after different treatments. Statistical p values: *p < 0.05, **p < 0.01, ***p < 0.001

To evaluate the biodistribution of BFE@BSA NPs within the body, 4T1 tumor-bearing BALB/c mice were established and tested. Fluorescent dye DiR labeled BFE@BSA NPs or free DiR were injected into tumor-bearing BALB/c mice, respectively. The mice were sacrificed to obtain tumors and main organs at designed time intervals (8, 12 and 24 h). As shown in Fig. 5A, quite weak DiR fluorescence intensity was observed and remained the same level at all the time points in the tumor from mice intravenously injected with free DiR. It was similar trend as previous reports, which was ascribed to its weak tumor retention. [52] Moreover, the liver and spleen were the main accumulation organs of free DiR. By contrast, stronger fluorescence intensity was observed in the tumor from mice treated with BFE@BSA NPs. In Fig. 5B, quantification of fluorescence intensity indicated that the accumulative fluorescence intensity at the tumor site increased with prolonging time and reached the maximum fluorescence intensity at 12 h post-injection (Additional file 1: Figure S10). The quantitative results of relative fluorescent ratio of tumor/liver displayed the similar trend (Fig. 5C). [53] It was worth mention that the accumulation of BFE@BSA NPs in the liver and spleen was significantly reduced, indicating that our nanomedicine was able to prolong the circulation lifetime of drugs and change metabolic pathways of drugs in the body. These results implied that BFE@BSA NPs could accumulate in the tumor region effectively.

### In vivo dose exploration of BLM

Even though BLM is an effective glycopeptide anticancer drug that could affect the cutting of single- and double-stranded DNA, the risk of pulmonary fibrosis caused by BLM is still not negligible. In order to achieve the highest anti-tumor activity of BLM while minimizing its potential side effects, before applying BFE@BSA NPs into further in vivo anti-tumor experiment, the optimal dosage of BLM was firstly explored. As the experimental scheme diagram of treatment shown in Fig. 5D, 4T1 tumor-bearing BALB/c mice were i.v. injected with PBS or BFE@BSA NPs with different concentrations (BLM: 2.5, 5 and 10 mg/kg) on day 5, 8 and 11. Both the body weight and tumor volume were measured every 2 days and tumors from mice with various treatments were separated and weighed on day 21 post-injection. As shown in Fig. 5E–H and Additional file 1: Figure S11A, compared with PBS, all the groups treated with BFE@BSA NPs exhibited anti-tumor effect (inhibition rate of 47.0% for 2.5 mg/kg, 61.7% for 5 mg/kg and 67.7% for 10 mg/kg) due to effective killing effect of nanoparticles on tumors.

According to the tumor growth curve, mice treated with BFE@BSA NPs (BLM: 5 mg/kg or 10 mg/kg) exhibited similar tumor-suppressive power, as no significant difference was observed both in the tumor volume and tumor weight. But when compared to mice treated with 2.5 mg/kg, better tumor inhibition with significantly smaller tumor size and low tumor weight was found in groups treated with 5 or 10 mg/kg. Then, the safety of nanomedicine was monitored by analyzing the weight of the mice and lung section. It was found that 10 mg/kg of BLM treatment gave rise to the obvious loss of body weight, while the weight of mice treated with 5 mg/kg remained unchanged. It might mean that BFE@BSA NPs (BLM: 10 mg/kg) had overrun the safe dose. The idea was further confirmed in the results of lung section by histological analysis. As shown in Fig. 5I and Additional file 1: Figure S11B, the extensive inflammation was observed in the group of 10 mg/kg, and this was also associated with widespread collagen (blue area by Masson’s trichrome staining) accumulation and alveolar structure disorder. While the group of 2.5 and 5 mg/kg presented mild inflammation, and there were no obvious pathological changes in lung structures. BFE@BSA NPs (BLM: 10 mg/kg) showed more severe pulmonary fibrosis of mice than other groups, which represented dose-dependent side effects. Therefore, the concentration of nanomedicine was set to BFE@BSA NPs (BLM: 5 mg/kg) in the following experiments.

### In vivo anti-tumor efficiency of synergistic therapy

**Fig. 6:**
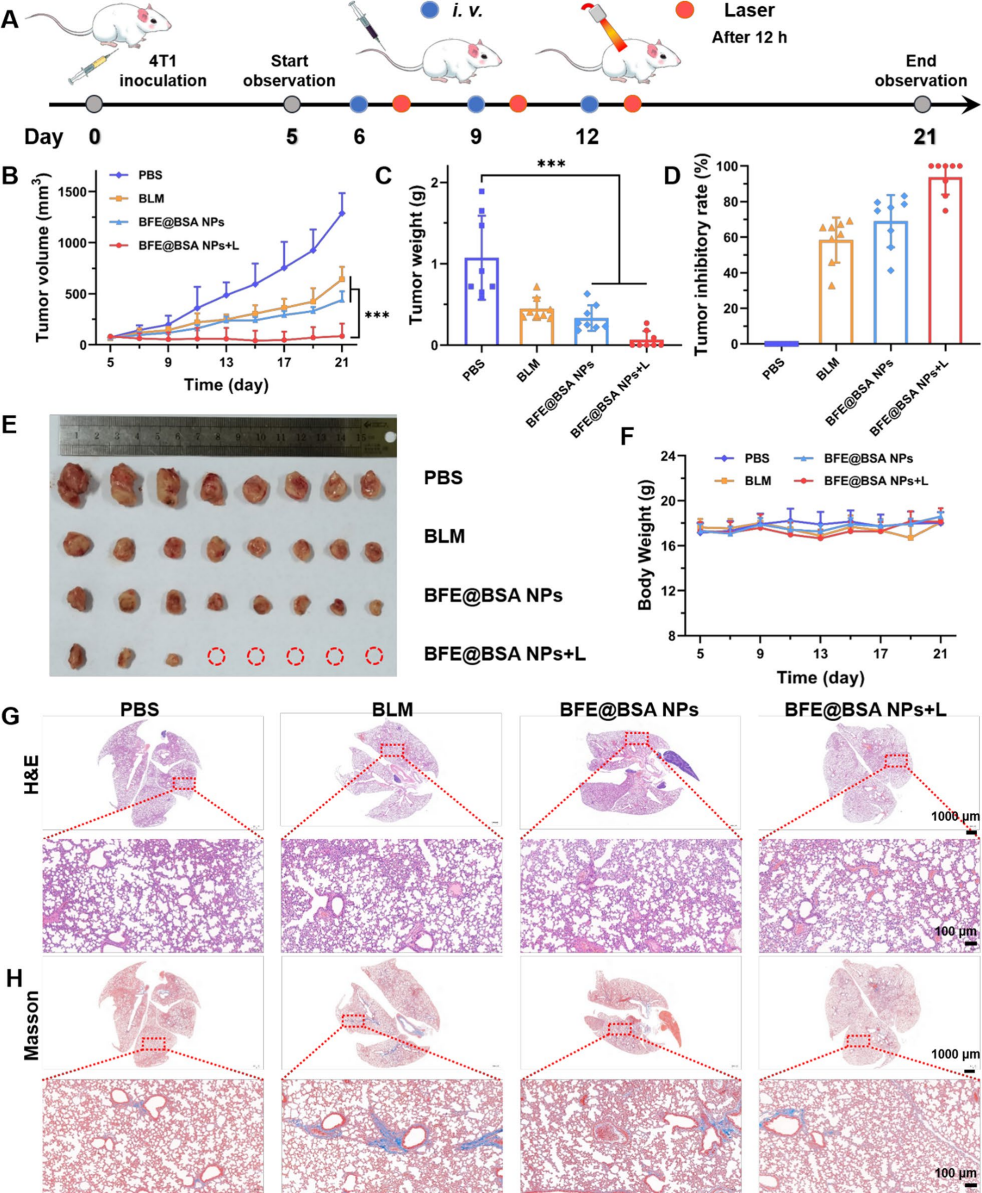
**A** Schematic diagram of 4T1 tumor treatment. The 4T1 tumor growth curve (**B**) tumor weight (**C**) and tumor inhibition rate (**D**) after different treatments (n = 8). **E** The photograph of tumors after different treatments. **F** The body weight of mice after different treatments. H&E (**G**) and masson’s (**H**) trichrome staining of the lung sections from the mice after different treatments. Statistical p values: *p < 0.05, **p < 0.01, ***p < 0.001

To further evaluate the combined therapeutic effects of CDT and PTT, 4T1 tumor-bearing BALB/c mice were i.v. injected with PBS, free BLM (5 mg/kg) or BFE@BSA NPs (BLM: 5 mg/kg) three times on day 6, 9 and 12. Half of the mice that received BFE@BSA NPs were further locally irradiated under NIR laser every 12 h-post injection at a power density of 2.5 W/cm^2^ for 2 min (Fig. 6A). Both the body weight and tumor volume were measured every 2 days and tumors and main organs from mice with various treatments were separated and weighed on day 21. As recorded in Fig. 6B–D and F, BLM and BFE@BSA NPs treatment presented moderate tumor inhibitory effect comparing to PBS group. The tumor inhibitory rate of BLM and BFE@BSA NPs were 58.4% and 69.0%, respectively. The superior anti-tumor efficiency of BFE@BSA NPs than free BLM was attributed to the enhanced accumulation at tumor site and the ROS amplification effect of nanomedicine. Beyond expectation, the BFE@BSA NPs + L group showed the eminent inhibition compared with other groups (tumor inhibition rate was calculated to be 93.7%) without obvious weight loss of mice. In Fig. 6E and Additional file 1: Figure S12, complete tumor ablation was achieved in five mice of all eight mice (5/8). During the entire experimental period, the therapeutic efficacy was maintained well and no recurrence was observed in the ablated tumor. These results demonstrated the superiority of combination therapy of chemotherapy, CDT and PTT. Histological analysis of the lung sections stained with H&E and Masson’s trichrome was conducted to assess the side effects of pulmonary fibrosis. As shown in Fig. 6G and H, severe inflammation was not observed in each group, and there were no obvious pathological changes in the lungs of all groups. In general, BFE@BSA NPs + Laser could achieve the desirable therapeutic effect with minimal toxic side effects.

## Conclusions

In summary, we constructed a metal-phenolic networks based theranostic nanoplatform through self-assembly and protein absorption. BFE@BSA NPs had great stability and photothermal conversion ability. Coordinate bonds were the driving force for the formation of BFE@BSA NPs, thus making the systems pH sensitive. Moreover, it’s found that BFE@BSA NPs could serve as photothermal transduction agents and T_1_-weighted MRI contrast agents. In vitro and in vivo study on triple-negative breast cancer model revealed BFE@BSA NPs could achieve potent combinational therapy of chemotherapy, CDT and PTT. MRI guided individualized precise therapy might shed new light on clinical cancer treatment.

## Supplementary Information

**Additional file 1:** Supporting information including additional figures and tables.

## Data Availability

All data generated or analyzed during this study are included in this article and its Additional file 1. The data used to support the findings of this study are available from the corresponding author upon reasonable request.

## Abbreviations

TNBC: Triple-negative breast cancer
MPNs: Metal-phenolic networks
BLM: Bleomycin
BSA: Bovine serum albumin
MRI: Magnetic resonance imaging
CDT: Chemodynamic therapy
ROS: Reactive oxygen species
TME: Tumor microenvironment
•OH: Hydroxyl radicals
PTT: Photothermal therapy
PTAs: Photothermal transduction agents
