# Supplementary material for: All-in-one approaches for triple-negative breast cancer therapy: metal-phenolic nanoplatform for MR imaging-guided combinational therapy
Source: J Nanobiotechnology. 2022 May 12;20:226. doi: 10.1186/s12951-022-01416-7 (PMC9097361; doi:10.1186/s12951-022-01416-7)
Supplement: Supplementary file 1 — Additional file 1: Supporting information including additional figures and tables. [file 12951_2022_1416_MOESM1_ESM.docx]

**Additional file 1 for**

**All-in-One Approaches for Triple-negative Breast Cancer Therapy: Metal-phenolic Nanoplatform for MR Imaging-guided** **Combinational Therapy**

Qi Xie^1^, Shichao Li^2^, Xingxing Feng^1^, Jingyu Shi^3^, Yang Li^1^, Guanjie Yuan^2^, Conglian Yang^1^, Yaqi Shen^2^*, Li Kong^1^* and Zhiping Zhang^1,4,5^*

E-mail: yqshen@hust.edu.cn; [kongl@hust.edu.cn;](mailto:kongl@hust.edu.cn;) zhipingzhang@mail.hust.edu.cn

^1^Tongji School of Pharmacy, Huazhong University of Science and Technology, Wuhan 430030, China

^2^Department of radiology, Tongji Hospital of Tongji Medical College of Huazhong University of Science and Technology, Wuhan 430030, China

^3^Liyuan Hospital of Tongji Medical College of Huazhong University of Science and Technology, Wuhan 430030, China

^4^National Engineering Research Center for Nanomedicine, Huazhong University of Science and Technology, Wuhan, 430030, China

^5^Hubei Engineering Research Center for Novel Drug Delivery System, Huazhong University of Science and Technology, Wuhan, 430030, China


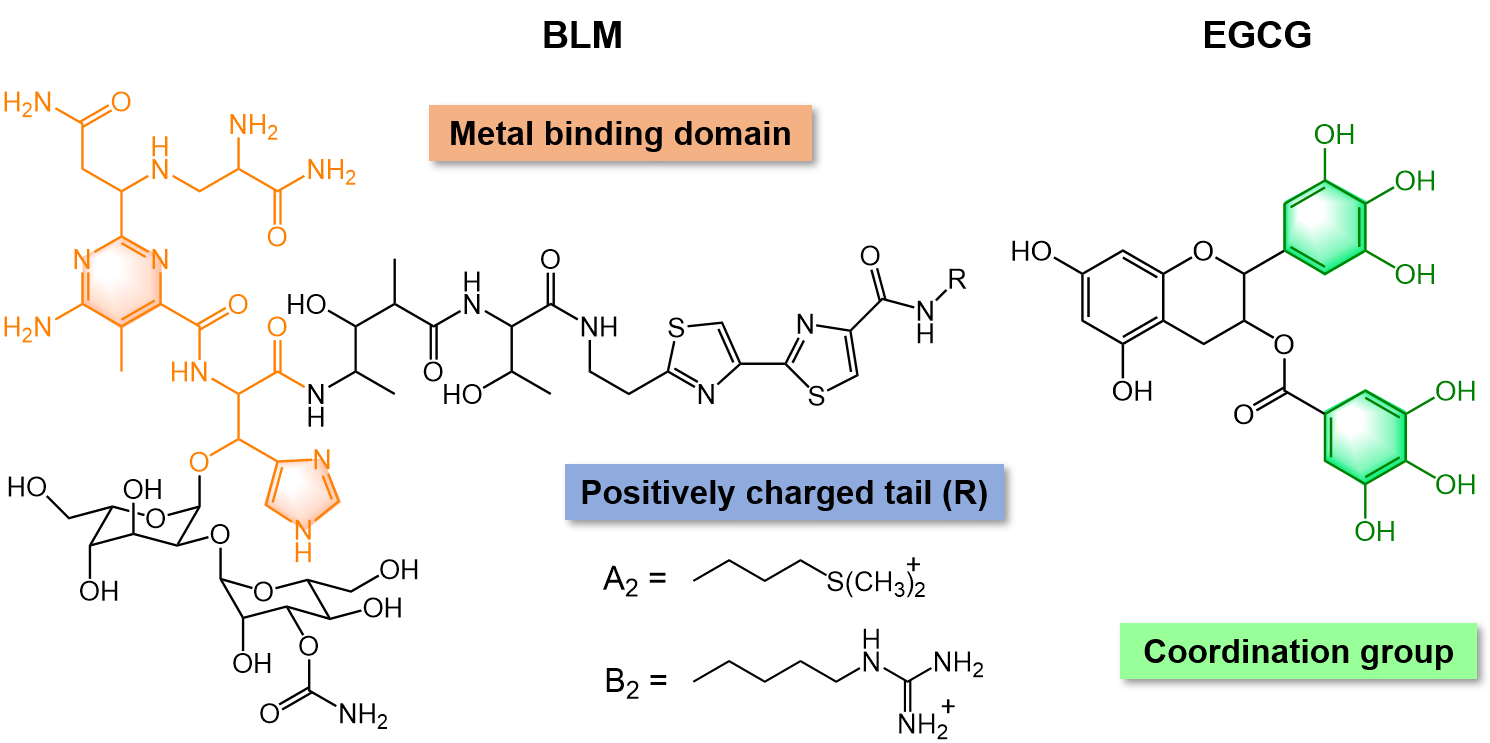


**Figure S1**: The chemical structure of BLM and EGCG. Bleomycin usually consists of two components differing in the positively charged tail —— bleomycin A2 and bleomycin B2.


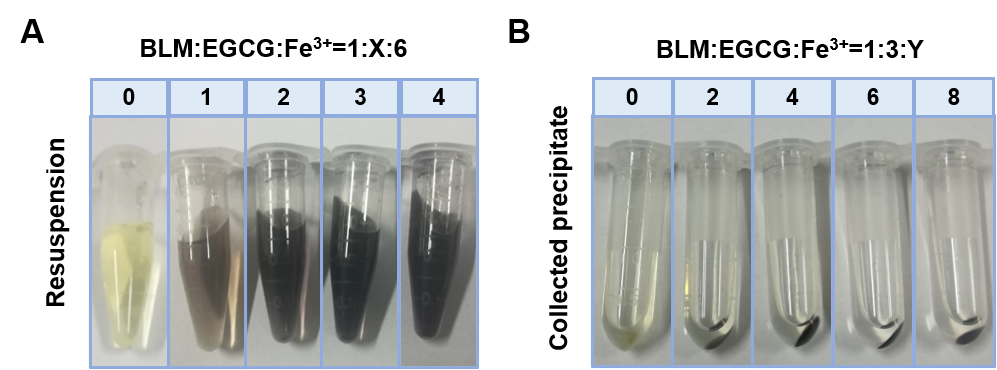


**Figure S2**: The photograph of BFE NPs at different molar ratios of EGCG (A) and Fe^3+^ (B). The nanoparticles were presented in the form of resuspension and collected precipitation, respectively.


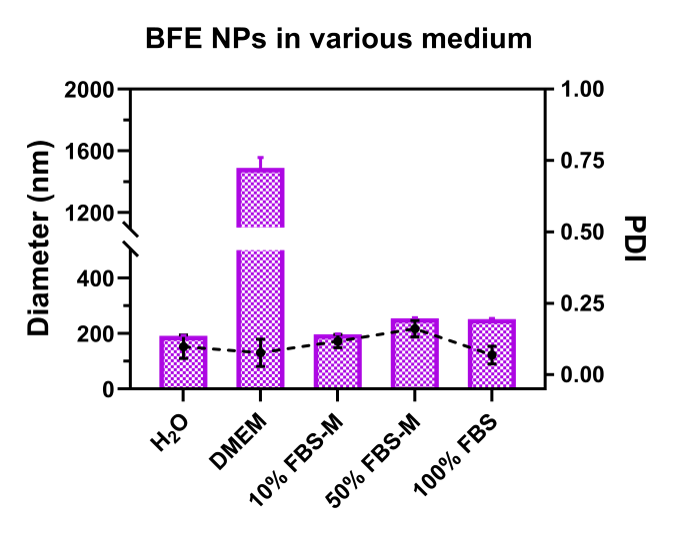


**Figure S3**: The particle size and PDI of BFE NPs in various media. There was also a lot of salt in the DMEM (cell culture medium).


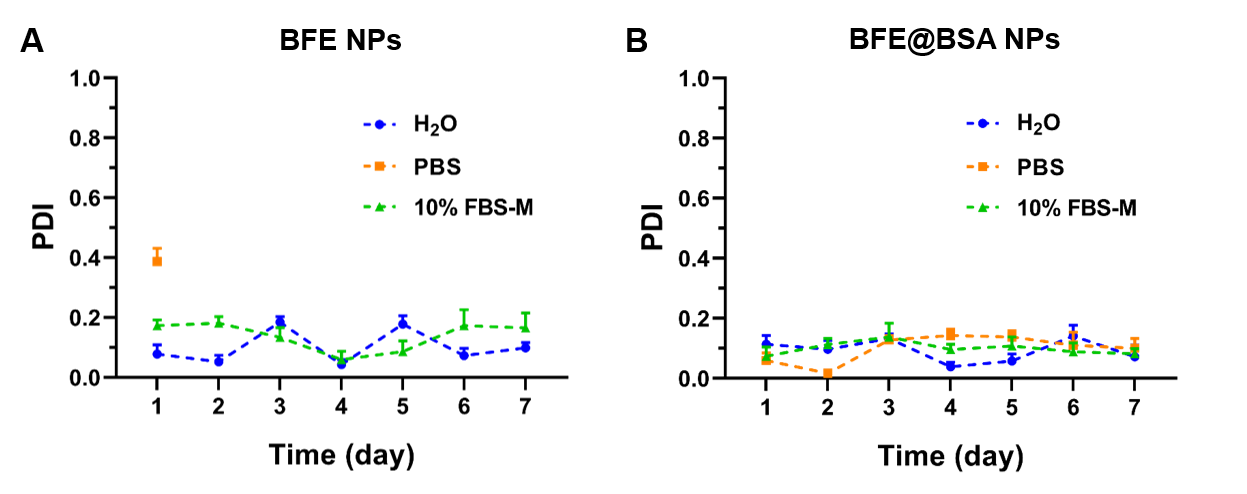


**Figure S4**: The long-term stability of BFE NPs and BFE@BSA NPs.


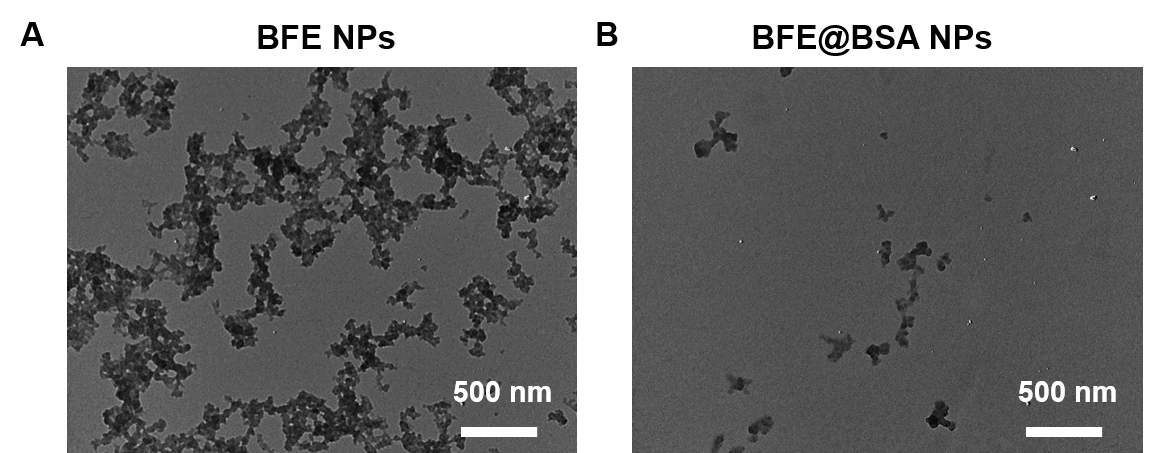


**Figure S5**: The TEM images of BFE NPs (A) and BFE@BSA NPs (B). Scale bars were 500 nm. (large field of view in Fig 1K and 1L).

**Table S1**: The drug encapsulation efficiency (EE) of BLM and Fe^3+^ in BFE NPs and BFE@BSA NPs.

| EE (%) | BLM | | Fe^3+^ | |
| --- | --- | --- | --- | --- |
|  | Mean | SD | Mean | SD |
| BFE NPs | 80.43 | 6.43 | 94.85 | 1.94 |
| BFE@BSA NPs | 65.04 | 4.31 | 77.21 | 4.72 |

The EE was calculated as follows: EE (%) = (Mass of drug in nanoparticles/Mass of drug added) × 100%. (n = 5 independent samples)


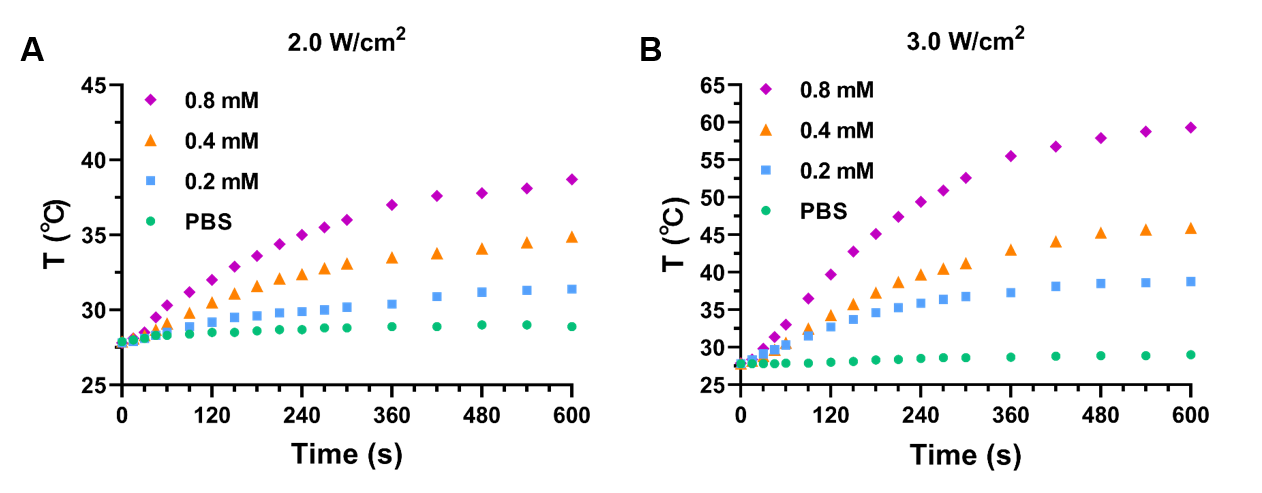


**Figure S6**: The temperature elevation of BFE@BSA NPs under laser irradiation (808 nm, (A) 2.0 and (B) 3.0 W/cm^2^) for 10 min at different Fe^3+^ concentrations (*Note: The trend of 0.8 mM in Figure S6A is the same one of 2.5 W/cm^2^ in Fig 2J; The trend of 0.8 mM in Figure S6B is the same one of 3.0 W/cm^2^ in Fig 2J*) .


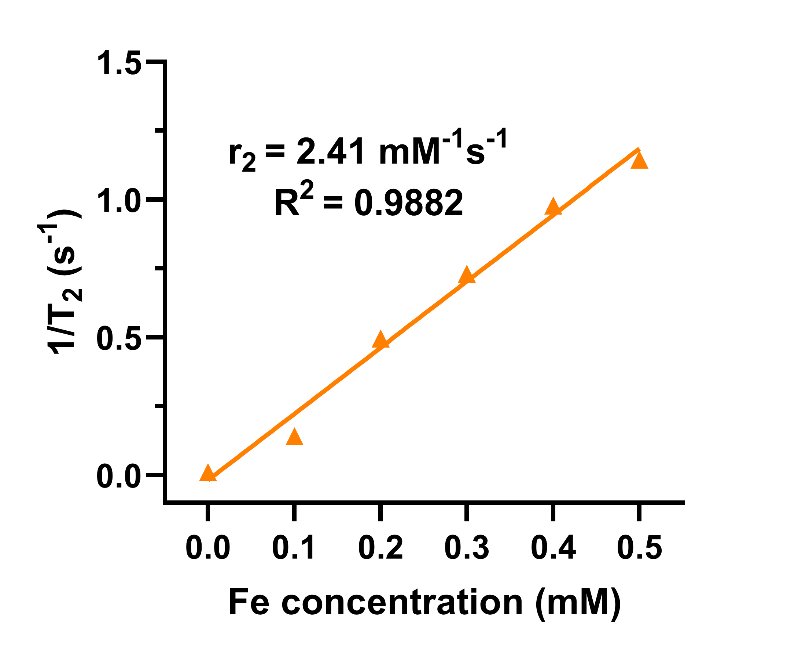


**Figure S7**: The linear fitting of 1/T_2_ of BFE@BSA NPs at different Fe^3+^ concentrations.


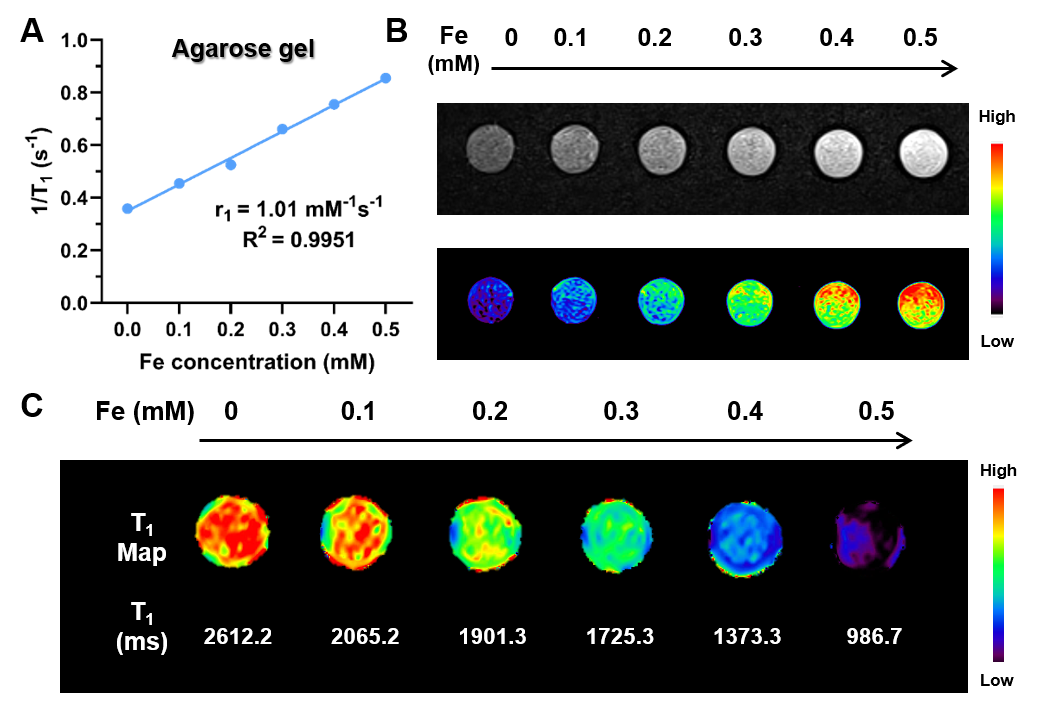


**Figure S8**: In vitro MRI study of BFE@BSA NPs in agarose gel. (A) The linear fitting of 1/T_1_ of BFE@BSA NPs at different Fe^3+^ concentrations. The T_1_-weighted MR images (B) and T_1_ mapping images (C) of BFE@BSA NPs nanoparticle in vitro.


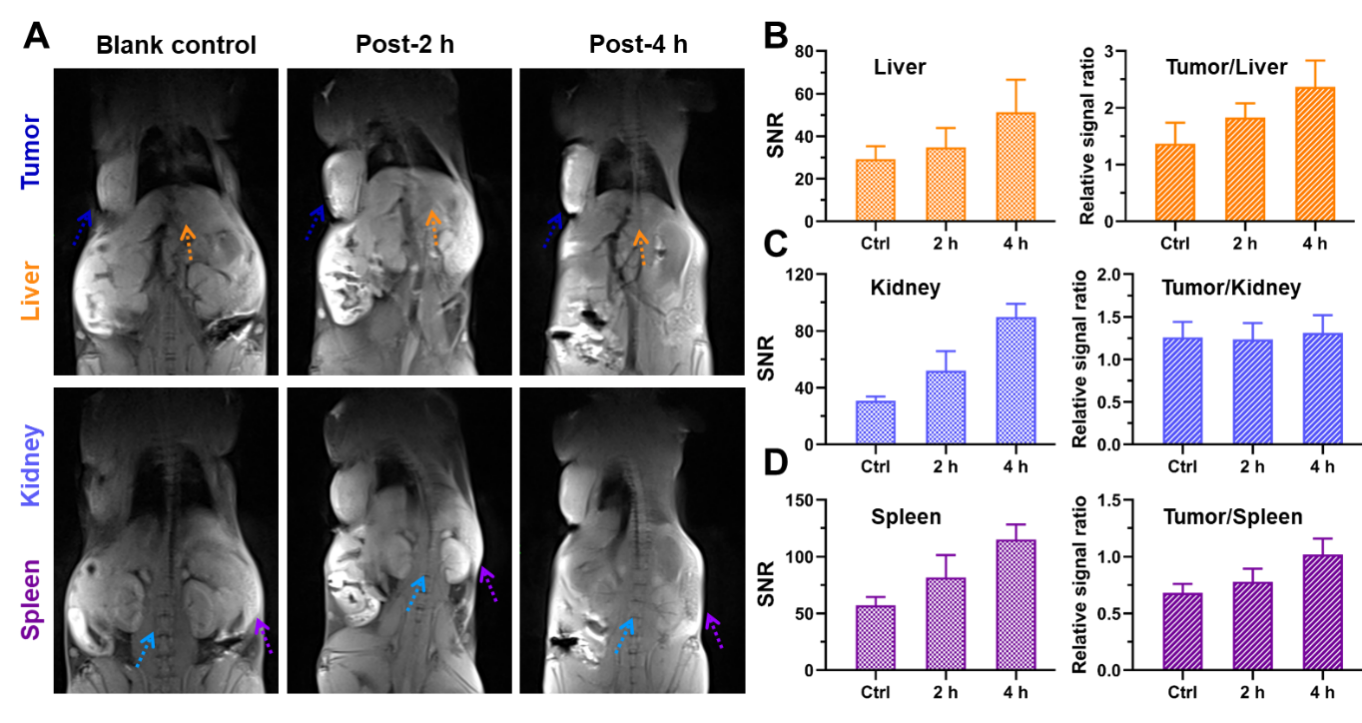


Figure S9: (A) The coronal T_1_-weighted MR images of different organs in the mice at different time points (the organs were marked with different colored arrows). (B-D) The corresponding SNR of liver, kidney and spleen and relative signal ratio of tumor to other organs (n = 3, different mice for each time point, *note: the mice in Figure S9 are the same mice in Fig 4J*).


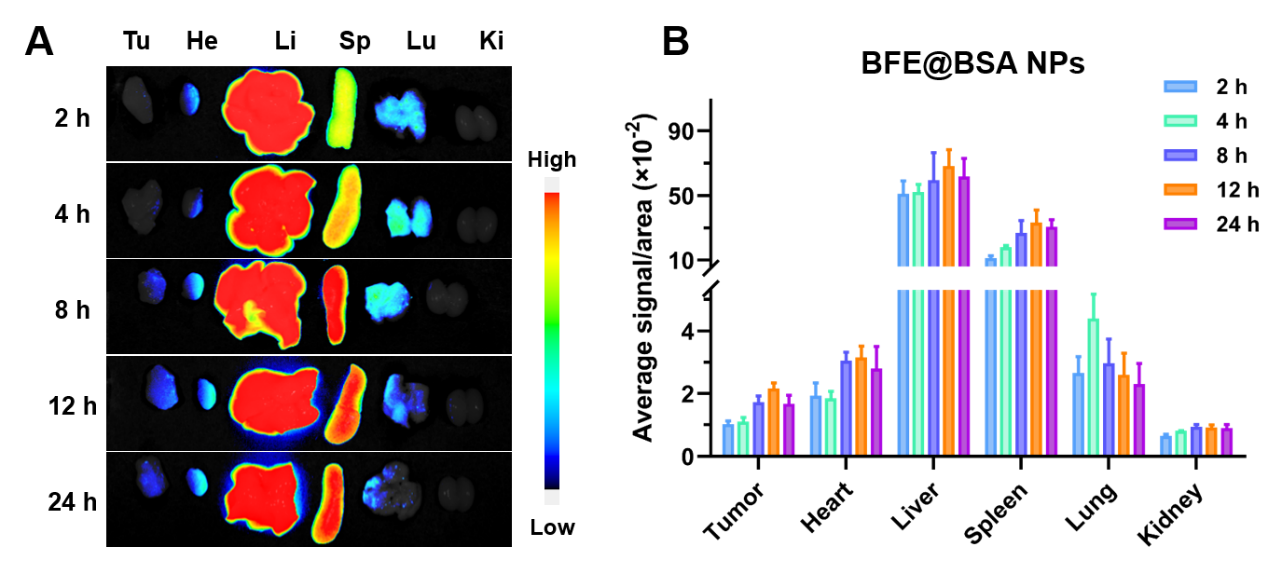


**Figure S10:** The fluorescence images (A) and quantitative analysis (B) of in vivo biodistribution of the DiR-loaded BFE@BSA NPs (n = 3, different mice for each time point).


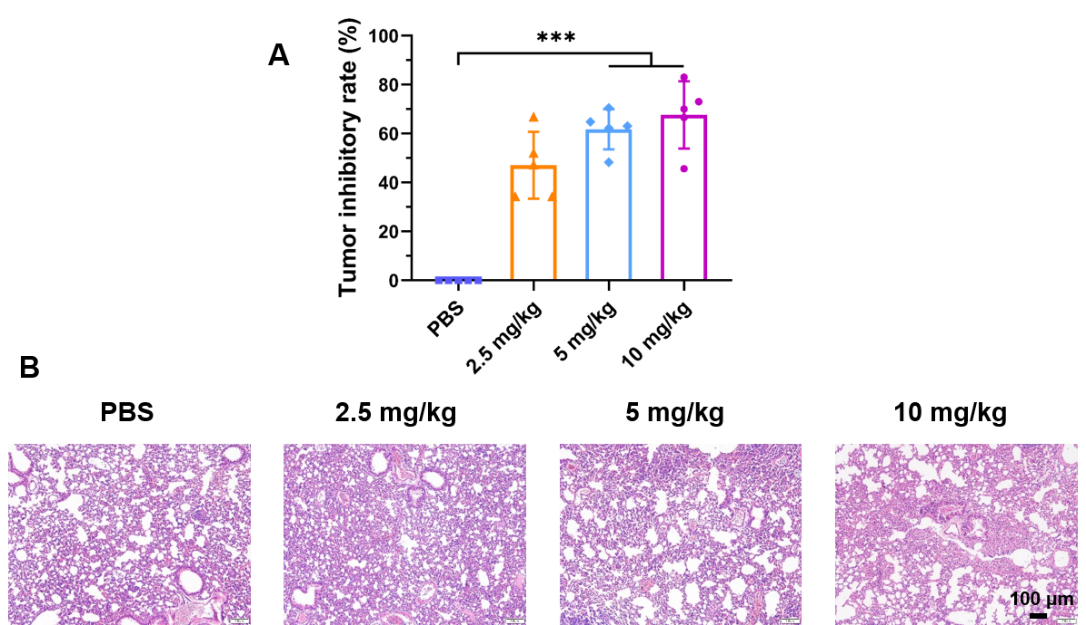


**Figure S11**: (A) The 4T1 tumor inhibition rate after different treatments (n = 5). (B) H&E staining of the lung sections from the mice after different treatments.


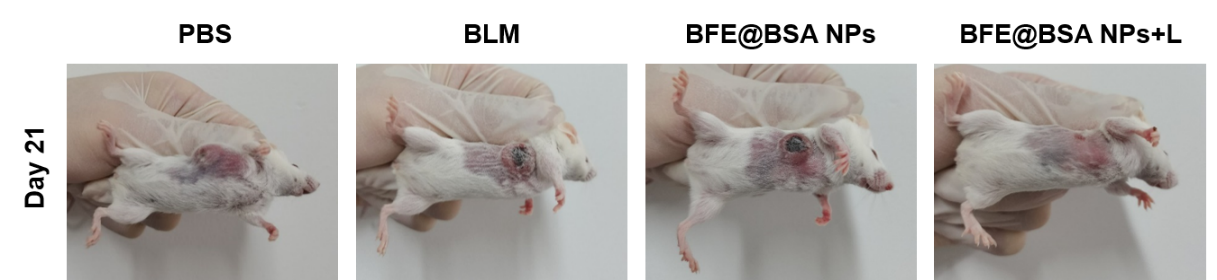


**Figure S12**: The 4T1 tumor of the mice after different treatments.
